# Network pharmacology to investigate the pharmacological mechanisms of muscone in Xingnaojing injections for the treatment of severe traumatic brain injury

**DOI:** 10.7717/peerj.11696

**Published:** 2021-07-20

**Authors:** Zhuohang Liu, Hang Li, Wenchao Ma, Shuyi Pan

**Affiliations:** 1 The Fifth Clinical Medical College of Anhui Medical University, Beijing, China; 2 Department of Hyperbaric Oxygen, Sixth Medical Center, Chinese PLA General Hospital, Beijing, China; 3 Department of Neurology, Sixth Medical Center, Chinese PLA General Hospital, Beijing, China

**Keywords:** XNJI, Muscone, Protein targets, Network analysis, Traumatic brain injury

## Abstract

**Background:**

Xingnaojing injections (XNJI) are widely used in Chinese medicine to mitigate brain injuries. An increasing number of studies have shown that XNJI may improve neurological function. However, XNJI’s active ingredients and molecular mechanisms when treating traumatic brain injury (TBI) are unknown.

**Methods:**

XNJI’s chemical composition was acquisited from literature and the Traditional Chinese Medicine Systems Pharmacology (TCMSP) database. We used the “absorption, distribution, metabolism, and excretion” (ADME) parameter-based virtual algorithm to further identify the bioactive components. We then screened data and obtained target information regarding TBI and treatment compounds from public databases. Using a Venn diagram, we intersected the information to determine the hub targets. Cytoscape was used to construct and visualize the network. In accordance with the hub proteins, we then created a protein–protein interaction (PPI) network using STRING 11.0. Gene Ontology (GO) and the Kyoto Encyclopedia of Genes and Genomes (KEGG) pathways were analyzed according to the DAVID bioinformatics resource database (ver. 6.8). We validated the predicted compound’s efficacy using the experimental rat chronic constriction injury (CCI) model. The neuronal apoptosis was located using the TUNEL assay and the related pathways’ hub proteins were determined by PCR, Western blot, and immunohistochemical staining.

**Results:**

We identified 173 targets and 35 potential compounds belonging to XNJI. STRING analysis was used to illustrate the protein–protein interactions and show that muscone played a fundamental role in XNJI’s efficacy. Enrichment analysis revealed critical signaling pathways in these components’ potential protein targets, including PI3K/AKT1, NF-kB, and p53. Moreover, the hub proteins CASP3, BCL2L1, and CASP8 were also involved in apoptosis and were associated with PI3K/AKT, NF-kB, and p53 signaling pathways. We showed that muscone and XNJI were similarly effective 168 h after CCI, demonstrating that the muscone in XNJI significantly attenuated neuronal apoptosis through the PI3K/Akt1/NF-kB/P53 pathway.

**Conclusion:**

We verified the neuroprotective mechanism in muscone for the first time in TBI. Network pharmacology offers a new approach for identifying the potential active ingredients in XNJI.

## Introduction

Traumatic brain injury (TBI) is caused by external mechanical forces including rapid acceleration, explosive waves, squeezing, and impact (Xiong, Mahmood & Chopp, 2013). Patients with severe TBI show cognitive impairment and physical deficits and may develop a long-term disability (Slavoaca et al., 2020). The Centers for Disease Control (CDC) estimated that at least 56,800 people die from TBI or its subsequent effects; 56-221 billion US dollars were spent annually on treating TBI (Oberholzer & Müri, 2019). The cost of treatment for TBI and its poor prognosis creates an urgent need for new and effective treatments.

A growing number of traditional Chinese medicines (TCM) are being applied to eliminate diseases and the clinical treatment of TBI should be explored in light of the philosophy of TCM (Xu et al., 2014). Studies have shown that Xingnaojing injection (XNJI) played a significant role in scavenging free radicals, reducing endogenous pyrogens in cerebrospinal fluid, inhibiting ischemia-reperfusion-induced apoptosis of neural cells, reducing brain edema, and improving circulation (Ma et al., 2020; Qu et al., 2019; Zhang et al., 2020; Zhang et al., 2018). Compounds in XNJI including geniposide and borneol have anti-inflammatory and neuroprotective properties in rats and septic mice with TBI (Wang et al., 2019; Yuan et al., 2020). Previous network pharmacology research has reported that XNJI can also protect against brain injury in AD (alzheimer disease) and stroke (Chen et al., 2018; Wang et al., 2020). Furthermore, some Chinese studies on XNJI demonstrated a favorable effect in TBI (Yan, 2011; Song et al., 2018). Systemic pharmacology has been widely used to explore its multi-component, multi-target, and multi-pathway mechanisms of action (Hopkins, 2007). The underlying molecular mechanism and potential active compounds of XNJI in treating TBI, however, remain unknown.

In this study, the network was constructed according to screening hub target proteins of TBI and active chemical compounds in XNJI.By combining functional enrichment and PPI analysis, we obtained a presumed bioactive compound (muscone). Finally, muscone, a predicted compound, was identified and first validated through in a TBI-induced animal model in this study. This study investigates molecules’ mechanisms by constructing a “drug-component- target-disease” network (Zhou et al., 2020) and presented a more comprehensive treatment concept through the integration of bioinformatics tools (Li & Zhang, 2013; Li et al., 2007).

## Methods & Materials

### Reagents

XNJI was obtained from Jiminkexin Pharmaceutical Company (Wuxi, China). The number of China Food and Drug Administration was Z32020562. Muscone and LY294002 (PI3K inhibitor) were obtained from Sigma (St. Louis, MO, USA). We obtained antibodies against p-65, p65, p-PI3K, PI3K, p-Akt1 (Thr308), Akt1, Bax, Bcl-2, cleaved caspase-3, p53, and *β*-actin from Abcam Biotechnology (Abcam, Cambridge, MA, USA).

### Acquisition of chemical ingredients and evaluation of pharmacokinetics

The XNJI ingredients are collected based on previous studies and the Traditional Chinese Medicine System Pharmacology Database (TCMSP) (https://tcmspw.com/tcmsp.php) (Ru et al., 2014), the Bioinformatics Analysis Tool for Molecular mechanism of Traditional Chinese Medicine (BATMAN-TCM) (Liu et al., 2016b), the Encyclopedia of Traditional Chinese Medicine (ETCM) (Xu et al., 2019), PubChem (https://pubchem.ncbi.nlm.nih.gov/), and SwissADME (http://www.swissadme.ch/) to determine the components of XNJI. These resources provided the names or structure of specific compounds. We used the ADME parameter-based virtual algorithm to identify bioactive components with a drug-likeness threshold ≥ 0.18 (Liu et al., 2016a) and barrier permeability of ≥0.30 (Li et al., 2015). Besides, some molecules in the previous studies that have been proven to pass through the blood–brain barrier will be included.

### Screening bioactive component protein targets

The active ingredient targets in XNJI were obtained from the TCMSP target section. We used Swisstarget Prediction for target prediction if our search did not return results (http://www.swisstargetprediction.ch/). TBI-related target genes were obtained from DisGeNET (http://www.disgenet.org/), Online Mendelian Inheritance in Man (OMIM, https://omim.org/), and the GeneCards database (https://www.genecards.org/). We removed any duplicates from the previous search results. The genes overlapping the compounds and target genes of TBI were included in a Venn diagram using a bioinformatics tool (http://www.bioinformatics.com.cn/).

### Constructing the compound-target network

We used Cytoscape ver. 3.7.2 (https://cytoscape.org/) to construct and visualize the network. Nodes were used to show target proteins or molecules, and edges indicated the correlations between targets and compounds. Larger nodes indicated more links.

### Construction of the protein interaction network

We used STRING 11.0 (http://string-db.org/cgi/input.pl) to establish the network of TBI-related PPI (Szklarczyk et al., 2017). The top 30 hub proteins were screened for analysis.

### Gene ontology and pathway enrichment analysis

GO and KEGG functions were analyzed using the DAVID Bioinformatics Resources database (ver. 6.8) to illustrate the overlap (https://david.ncifcrf.gov/) (Huang da, Sherman & Lempicki, 2009). We created bar and bubble charts of GO and KEGG using R (version 4.0.2).

### TBI model

Sprague-Dawley (SD) rats, 250–300 g, were provided by the Cancer Institute of the Chinese Academy of Medical Science (Beijing, China). All animals were handled following the ethical guidelines for animal-related experiments (ethical approval number: 2013022177). The experimental procedures were approved by the Animal Ethics Committee of the Chinese PLA General Hospital. Rats fasted 6–8 h before the operation. Our model was constructed following the controlled cortical impact (CCI) method (Romine, Gao & Chen, 2014; Schwulst & Islam, 2019) and SD rats were anesthetized by aspiration using isoflurane (velocity of flow 7-8L/min, concentration 3%). The craniotomy was performed midway between Bregma (*x* = 0 mm, *y* = 0 mm, *z* = 0 mm, after calibration) and lambda (−0.3 mm <z <0.3 mm) on the left side; the location of the craniotomy (*x* = 3 mm, y = −3.5mm) was lateral to the midline. Skull slices were then removed with the dura undisturbed. The CCI was applied perpendicular to the surface of the brain via PinPoint™ Precision Cortical Impactor (RWD, Shenzhen, China) with an impact tip measuring five mm in diameter, 4 m/s impact velocity, 500 ms impact duration time, and six mm depth. The craniotomy was sealed using bone wax and then the scalp incision was disinfected and sutured.

Part 1:36 SD rats were categorized into six arbitrary groups, which were as follows: TBI at 24, 72, and 168 h after injury, sham, TBI + XNJI, and TBI + muscone for RT-PCR. Part 2:48 SD rats were categorized into four groups randomly (sham, TBI + muscone, TBI, and TBI + muscone + LY294002 (PI3K inhibitor)) at 168 h for Western blot and apoptosis detection (Fig. S1).

### Drug administration

XNJI was injected intravenously 10 ml/kg daily (Yang et al., 2015), and muscone was administered at 0.54 mg/kg/d equivalently according to the manufacturer’s instructions. The muscone injection was administrated with LY294002 (10 uL, 100 nmol in 25% DMSO of PBS) in the TBI + muscone + LY294002 group (Ge et al., 2017). The other groups received the same volume (Fig. S1).

### Terminal deoxynucleotidyl transferase-mediated dUTP nick-end labelling (TUNEL) assay

Cellular apoptosis was identified based on a TUNEL assay according to the manufacturer’s protocol (Meilunbio, Dalian, China). Slides were prepared by dewaxing, hydration, washing, and penetration. A 50 ul Tunel detection solution was added and the slides were washed with PBS and incubated for 60 mins. Finally, glass substrates were sealed with an anti-fluorescence attenuation solution. Fluorescence quantitative analysis was completed using Image J (National Institutes of Health, Bethesda, MD, USA).

### Immunohistochemical (IHC) staining

IHC analysis was performed as described in a previous study (Zhang et al., 2018).

### Western blot

Ten-thousand gram The TBI rat Cerebral cortex was lysed in RIPA buffer for 30 min and subsequently centrifuged for 5 min at 4 °C 10,000 g, to get proteins. The generated protein was separated using 10% SDS gel. The resulting proteins were transferred electrophoretically from SDS–PAGE to PVDF membranes with a wet-blot transfer apparatus (Bio-Rad, California, USA). The membranes were incubated overnight at 4 °C with p53, cleaved caspase3, p-p65, p65, PI3K, Akt1, and p-Akt1 (Thr308), and p-PI3K antibodies, followed by a second antibody marked with horseradish-peroxidase for 90 min at room temperature. Bands were visualized with an improved chemiluminescence kit following the manufacturer’s protocol and were detected with a Bio-Rad-based imaging system (Bio-Rad, Hercules, CA, USA). We analyzed densitometry quantitation using Image J.

### RT-PCR

RNA was extracted from the affected brain cortex using TRIzol^®^ reagent (Invitrogen Life Technologies, Paisley, Scotland). All RNA (4 ul) was changed into complementary DNA (cDNA) in a reverse manner using M-MLV Reverse Transcriptase (Invitrogen, Paisley, Scotland). The cDNA was incubated using the Fast SYBR^®^Green Master Mix Bulk Pack (Invitrogen, Paisley, Scotland) for quantitative fluorescence analysis. The primer sequence was as follows: BCL-2, forward primer, 5′ -CCGGGAGAT CGATGAAGTA-3′, reverse primer, 5′-CATATTTGTTTGGGGCA TGTCT-3′; Bax, forward primer, GTGAGCGGCTGCTTGTCT, reverse primer, GTGGGGGT CCCGAAGTAG; Caspase 3, forward primer, AATTCAAGGGACGG GTCATG, reverse primer, GCTTGTGCGCGTACAGTTTC; GAPDH, forward primer, ACAGCAACAGGGTGGTGGAC, reverse primer, TTTGAGGGTGCAGCGAACTT.

Forty amplification cycles (pre-denaturation at 94 °C for 2 min, denaturation for 30 s at 94 °C, and annealing for 30 s at 62 °C) were applied in the amplification reaction analysis using Rotor-Gene Q PCR’s detection system (Qiagen Biosystems, CA, USA).

### Statistical analysis

All data are shown as mean ± standard deviation (SD). The statistical significance was analyzed using one- or two-way ANOVA in Graphpad Prism 8; statistical significance was *P* < 0.05.

## Results

### Screening active molecules in XNJI

XNJI compounds were obtained from earlier studies and public databases. The BBB permeability and drug likeness’s threshold values were set greater than 30% and 0.18, respectively, to screen for active ingredients in the databases. In Moschus, cholesterol, n-nornuciferine, 17-beta-estradiol, cholesteryl ferulate, and muscone supported the established pharmacokinetic standard. For Radix-Curcumae β-sitosterol, sitosterol, Oxy-curcumenol, 1,7-Diphenyl-3-acetoxy-6(E)-hepten, 4,5-Epoxy-12-acetoxy-7a,11a-dihydrogermacradien-8-one, difurocumenone, caryophyllene, Germacron, curcumin, curdione, and curzerenone were active compounds. Caryophyllene was shared with Curcumae-Radix and Borneolum-Syntheticum. There were seven active ingredients in Borneolum-Syntheticum with the exception of caryopyllene. Radix Curcumae and Fructus Gardeniae both have beta-sitosterol. Fructus Gardeniae possesses 13 active compounds including beta-sitosterol. We were able to obtain 35 active molecules based on the ADME parameters in the TCMSP database and previous studies. We then used PubChem to produce 2D structures for the next analysis.

### Active ingredients and TBI target predictions

The targets of the active ingredients in XNJI were acquired using TCMSP. Swisstarget Prediction identified other potential targets using 2D structures. The GeneCards, Disgenet, and Omit databases were used to retrieve disease-related targets. Duplicate targets were removed from our results. The gene name was transferred from the protein name using the UniProt database (http://www.UniProt.org/). Data in these databases are taken from the literature and are constantly updated to provide relatively comprehensive references.

### Constructing compound-target networks

**Figure 1:**
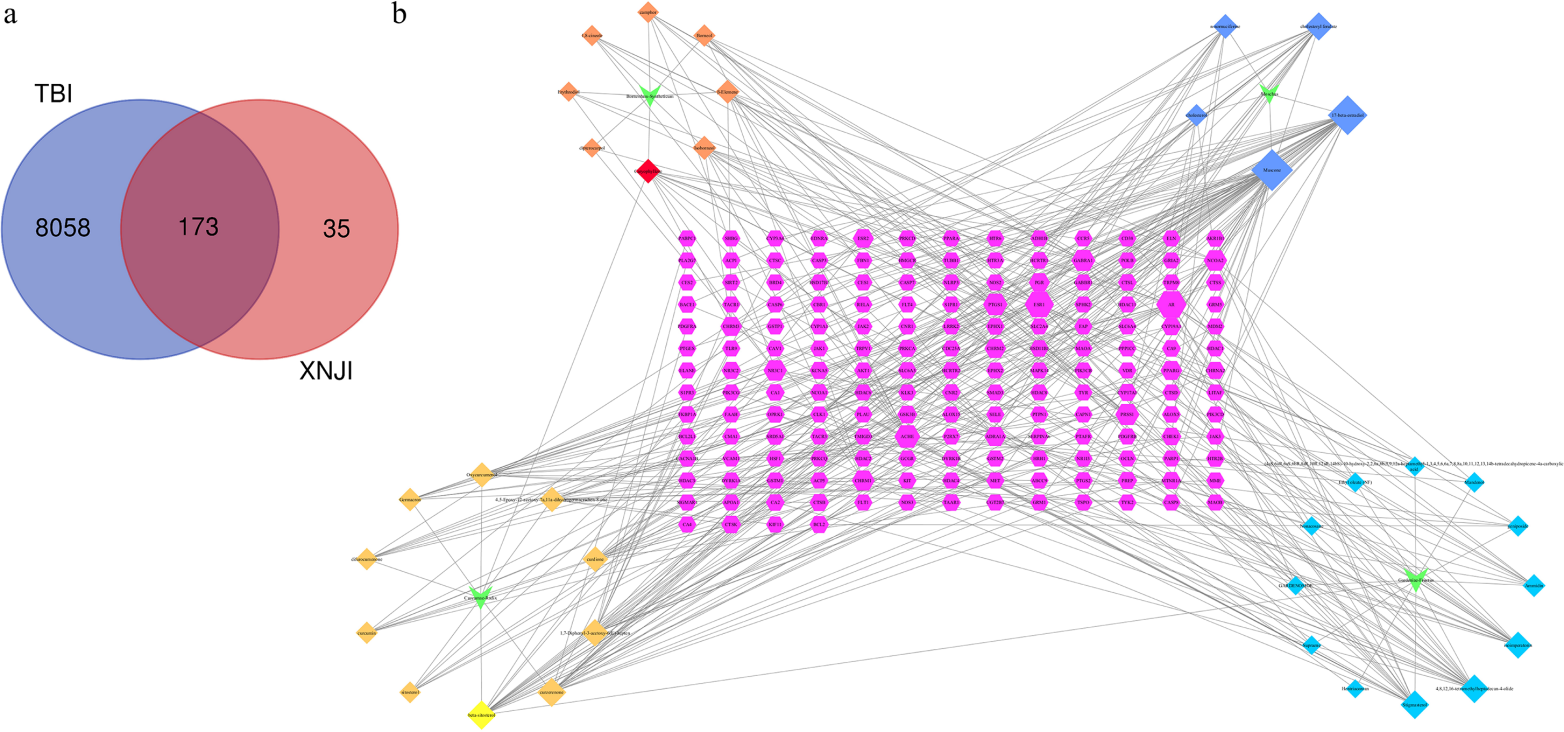
Compound-target identification. (A) The Venn diagram of 173 potential common targets (B) This network represents a global view of the potential compounds (diamonds) and targets (hexagons) in XNJI, and it comprised 228 nodes (35 potential compounds and 173 potential targets) and 367 edges (compound-target interactions).

We retrieved 173 genes linked to TBI from the intersection of active ingredients and disease targets and used these to construct a Venn diagram (Fig. 1A). The network of compound-targets indicated that there were 173 target nodes, 35 compound nodes, and 404 edges (Fig. 1B). Larger nodes were used to indicate a greater degree of intersection to improve visualization. A larger degree in the network indicated the importance of the target or compound. The node with the largest degree value was muscone (degree = 42). It was much larger than the average value (3.81), indicating that it has potential therapeutic use in the treatment of TBI. Both AR (degree = 22) and ESR1 (degree = 20) played crucial roles in the network. Our analysis revealed that CASP3 (degree = 6), RELA (p65, a subunit of the NF-kB family, degree = 6), and BCL-2 (degree = 6) were larger than the average.

**Figure 2:**
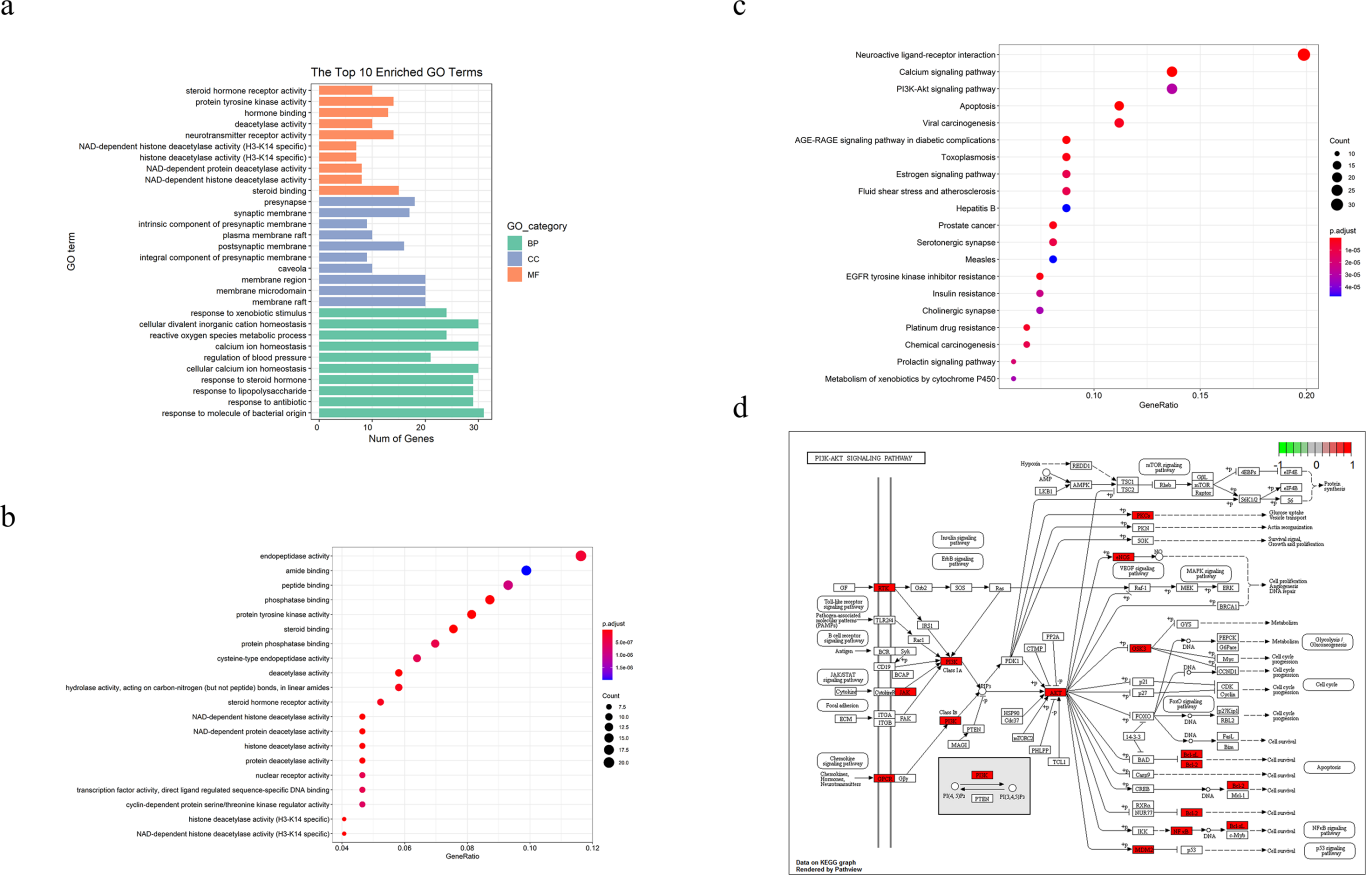
TBI-related Gene Ontology(GO), KEGG pathway of compound- target and diagram of possible treatment pathways. (A) The bar to describe *P*-adjust value range of top 10 GO enriched terms for each GO category(MF,CC,BP, P-adjust value < 0.01). (B, C) The dot plot of top20 enriched GO items and KEGG pathways (*P*-adjust value < 0.05). (D) The different biological processes are regulated by the XNJI’targets of PI3K/Akt(core pathway), NF-kB and p53 signaling pathways.

We performed GO and KEGG pathways enrichment analyses to verify the association between the predicted target and TBI. As shown in Figs. 2A and 2B, the GO terms (which include NAD-dependent histone deacetylase activity, steroid binding, membrane raft, membrane microdomain, response to molecule of bacterial origin, and response to antibiotics) were enriched in bar and bubble plots. We used an identical method to analyze potential targets in the critical pathway. The top 20 pathways were filtered and probably implicated in the signaling pathways of apoptosis, PI3K-Akt, p53 and the cell cycles, among others (Figs. 2C and 2D).

### PPI network and top 30 targets from the PPI network

**Figure 3:**
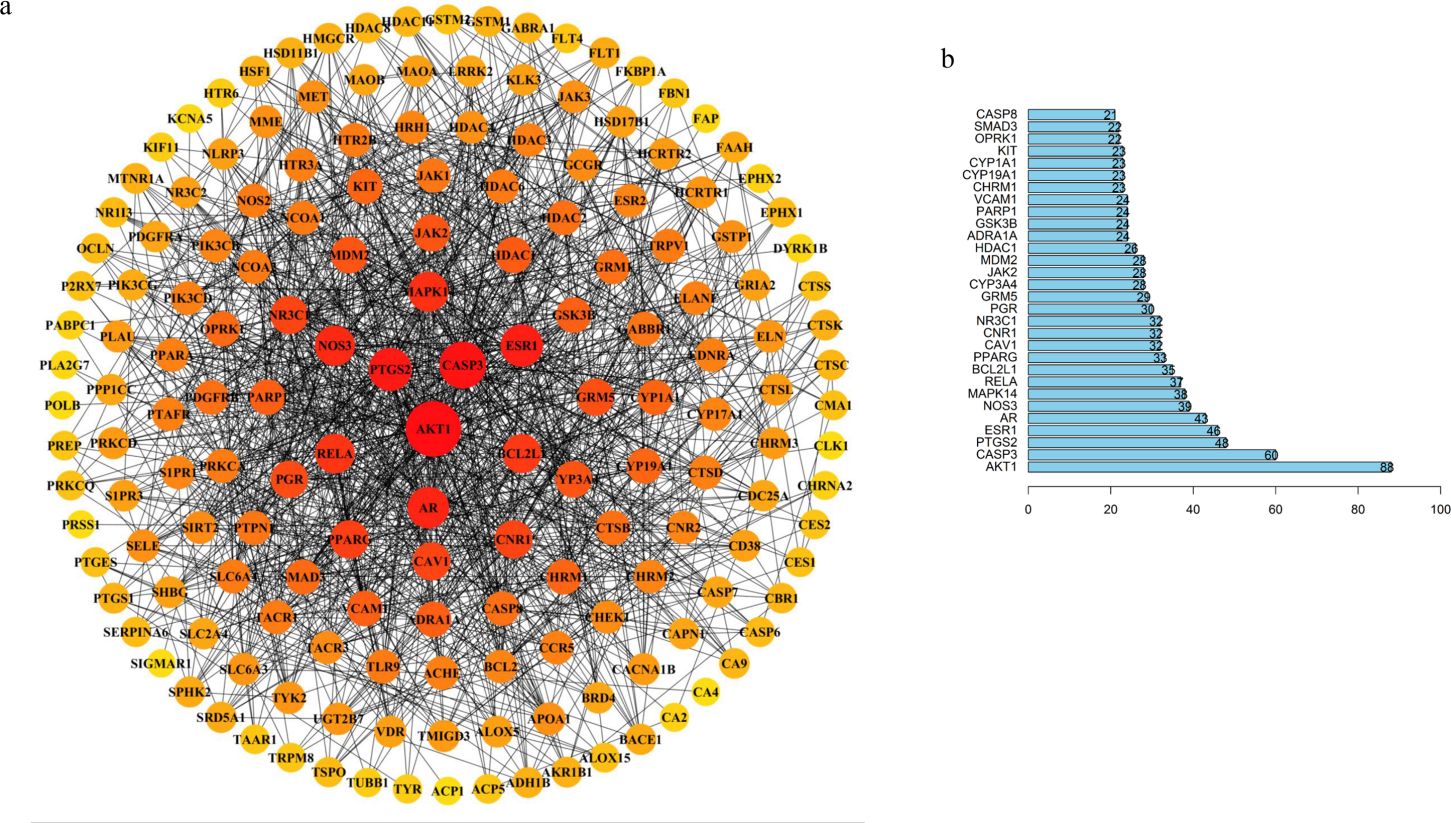
TBI-related PPI network and bar chart of top 30 targets. (A) Degree sorted circle layout of the PPI network visualized using STRING and Cytoscape software. (B) The length of the histogram represents the degree value of the genes.

We explored the hub proteins that were potential therapeutic targets in the PPI network to determine the actions of the molecular mechanisms in TBI. We removed the unrelated targets which left a total of 169 targets and 1,279 interactions in the PPI network (Fig. 3A). The linked targets were given a different color and size to represent the meaning of their interaction.

Our results showed that the five hub proteins AKT1, CASP3, RELA, BCL2L1, CASP8 (Degree: AKT1 = 88, CASP3 = 60, RELA = 37, BCL2L1 = 35, and CASP8 = 21; Betweenness: AKT1 = 6272.24553, CASP3 = 1679.86775, RELA = 486.18204, BCL2L1 = 537.55344, and CASP8 = 75.49232) were located at the core position. These were included in the top 30 key proteins that were visualized in a bar plot according to their degree in the PPI network (Fig. 3B). The nodes gradually become redder, bigger, and more centered according to their importance. We retrieved an interactive pathway from the KEGG Pathway database to determine the molecular mechanisms of the five proteins in TBI (Kanehisa et al., 2017). We found that these hub proteins may correlate with PI3K/Akt, p53 apoptosis, cell cycle, NF-kB, and glycolysis/gluconeogenesis (Fig. 2D). AKT1 is a critical regulator of apoptosis and cell survival in the central nervous system (Chen et al., 2018; Mehrafza et al., 2019); CASP3, BCL2L1 and CASP8 participate in the cell anti-apoptosis and apoptosis process (Ali et al., 2020; Loo et al., 2020; Sun & Pei, 2016); RELA is a crucial mediator in the NF-kB pathway and activates the survival and progression of cell (Cahill, Morshed & Yamini, 2016; Du et al., 2019; Gugliandolo et al., 2018). Muscone plays a critical role in the component-target network and animal experiments have shown that it can attenuate neuronal apoptosis (Wei et al., 2012). We found that AKT1 was targeted by muscone in PPI network. Meanwhile, muscone exhibited a high degree of matching with Akt1 (Fig. S4). Therefore, muscone may modulate neuronal apoptosis through the PI3K/Akt, NF-kB signaling pathway and p53 inhibition.

### Experimental verification

#### Temporal profiles of TBI-induced and XNJI-treated or muscone-treated changes in bax, BCL-2 and caspase 3, and p53 mRNA levels

**Figure 4:**
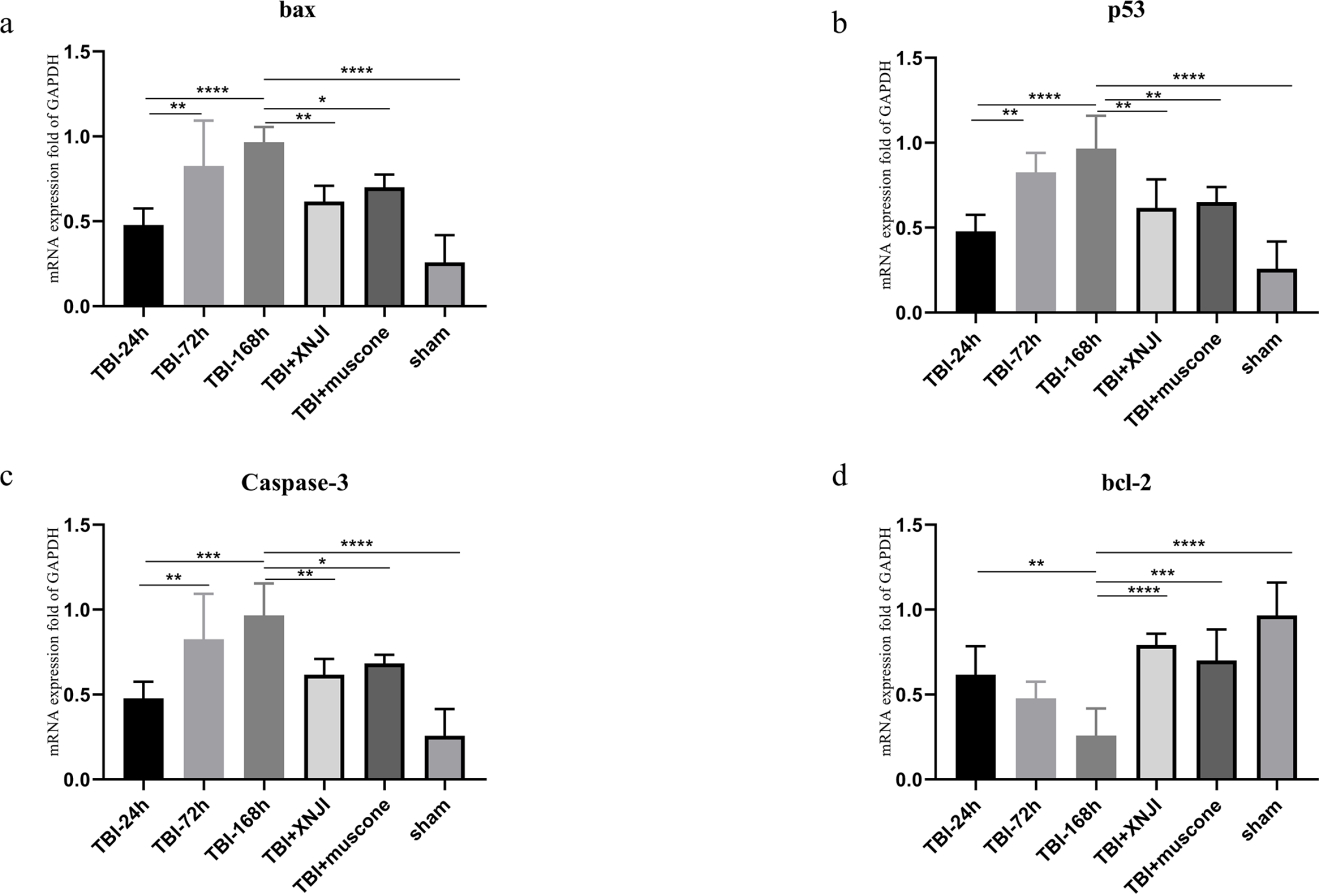
The time course of mRNA and the effect of XNJI and muscone on the expression of mRNA of the ipsilateral cerebral cortex 168 h after TBI. (A–D). The mRNA expression of bax, p53 and caspase-3 was significantly up-regulated 168 h after TBI while BCL-2 were down-regulated remarkably. The XNJI and muscone treatment down-regulated mRNA levels of bax, p53 and caspase 3 compared with the sham group, but BCL-2 had a reverse effect. *N* = 6, each group, ^∗^*P* < 0.05, ^∗∗^*P* < 0.01, ^∗∗∗^*P* < 0.001, ^∗∗∗∗^*P* < 0.0001.

The expression levels of BCL-2, bax, p53 and caspase 3 mRNA in the damaged cortex were investigated at a various range of times in TBI. As shown in Fig. 4, mRNA gradually increased 24 h after TBI up until 168 h after the damage occurred (Fig. 4) (*P* < 0.05). We detected mRNA expression levels at the 168 h mark in the TBI + XNJI, TBI + muscone, and sham groups. The results showed that these mRNA were remarkably down-regulated 168 h after treatment with XNJI and muscone, respectively (Fig.S3).

### Muscone affects neuronal apoptosis in the injured cerebral cortex 168 h after TBI

**Figure 5:**
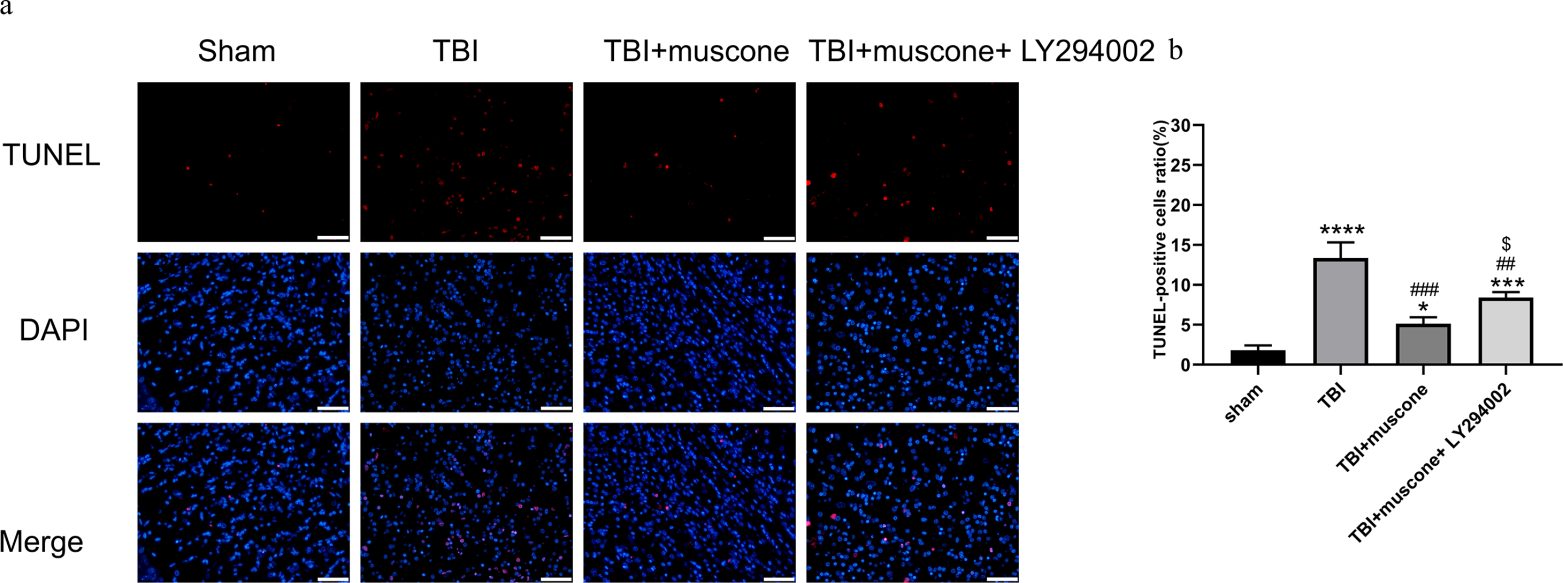
Cortical cellular apoptosis. (A) Representative TUNEL/DAPI photo- micrographs of the ipsilateral cortex in different groups (scale bar = 50 um). Colors of fluorescence: DAPI: blue; TUNEL: red. (B) Quantitative comparison of tunel -positive cells in the Sham, TBI, TBI+Muscone and TBI+Muscone+ LY294002 groups. *N* = 3, each group, ^∗^*P* < 0.05, ^∗∗∗^*P* < 0.001, ^∗∗∗∗^*P* < 0.0001 vs sham group; ^##^*p* < 0.01, ^###^*p* < 0.001 vs TBI group; *p* < 0.05 vs TBI+muscone group.

We used the TUNEL assay, IHC, and Western blot to explore the function of muscone in neuronal apoptosis. According to representative TUNEL staining, the red fluorescent spots indicated cellular apoptosis (Fig. 5A). The TUNEL-positive cells in the TBI group (11.4%–15.3%) exceeded those in the sham group (1.3%−2.5%) according to our quantitative results (Fig. 5B) (*P* < 0.05). Muscone treatment attenuated neuronal apoptosis 168 h post-TBI (4.3%−5.9%), and an injection of LY294002 (a specific inhibitor of PI3K) reversed the effect (7.9%−9.2%). This trend was observed in immunohistochemistry as well (Fig. S2).

### Muscone decreased Akt1 and PI3K phosphorylation in TBI

It has been shown that PI3K may activate and phosphorylate Akt1 (Yang et al., 2015). We used the Western blot to explore protein levels and determine how muscone affected the changes of Akt1/PI3K pathway.

**Figure 6:**
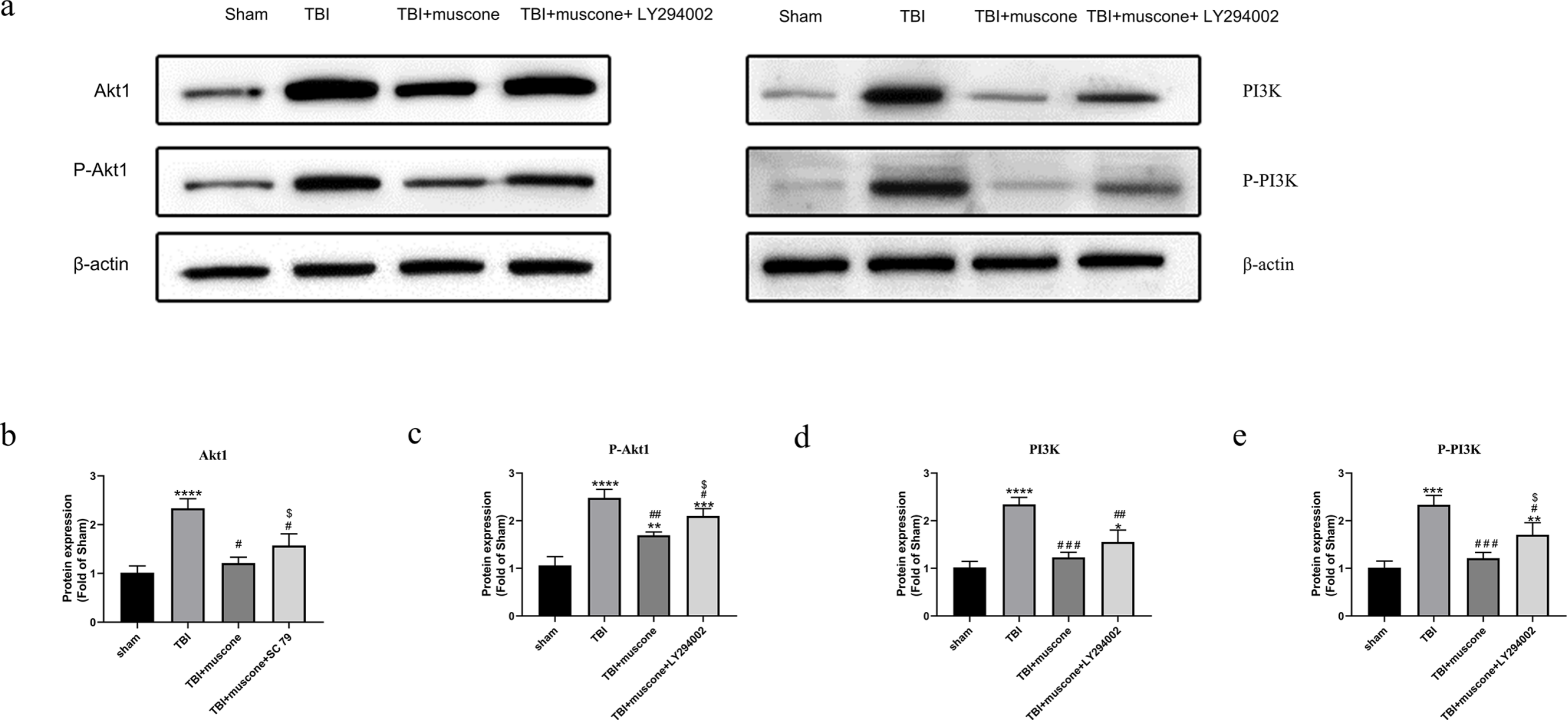
Muscone negatively regulates PI3K, phosphorylated PI3K, Akt1, phosphorylated Akt at Thr308 [p-Akt1 (Thr308)] of injured traumatic brain tissues in TBI rats. The expression levels were detected using the Western blotting analysis. Results are presented as mean± SD. *N* = 3, each group, ^∗^*P* < 0.05, ^∗∗^*P* < 0.01, ^∗∗∗^*P* < 0.001, ^∗∗∗∗^*P* < 0.0001 vs sham group; ^#^*p* < 0.05, ^##^*p* < 0.01, ^###^*p* < 0.001 vs TBI group; *p* < 0.05vs <TBI+muscone group.

The P-Akt1 level increased in the TBI group compared with the sham group; however, adding LY294002 counteracted the muscone’s protective effect. The phosphorylated PI3K(p-PI3K), Akt1, and phosphorylated Akt1 (p-Akt1, at the Thr308 site) protein expression were similar (Fig. 6).

### Muscone inhibited the activation of the NF-kB pathway

**Figure 7:**
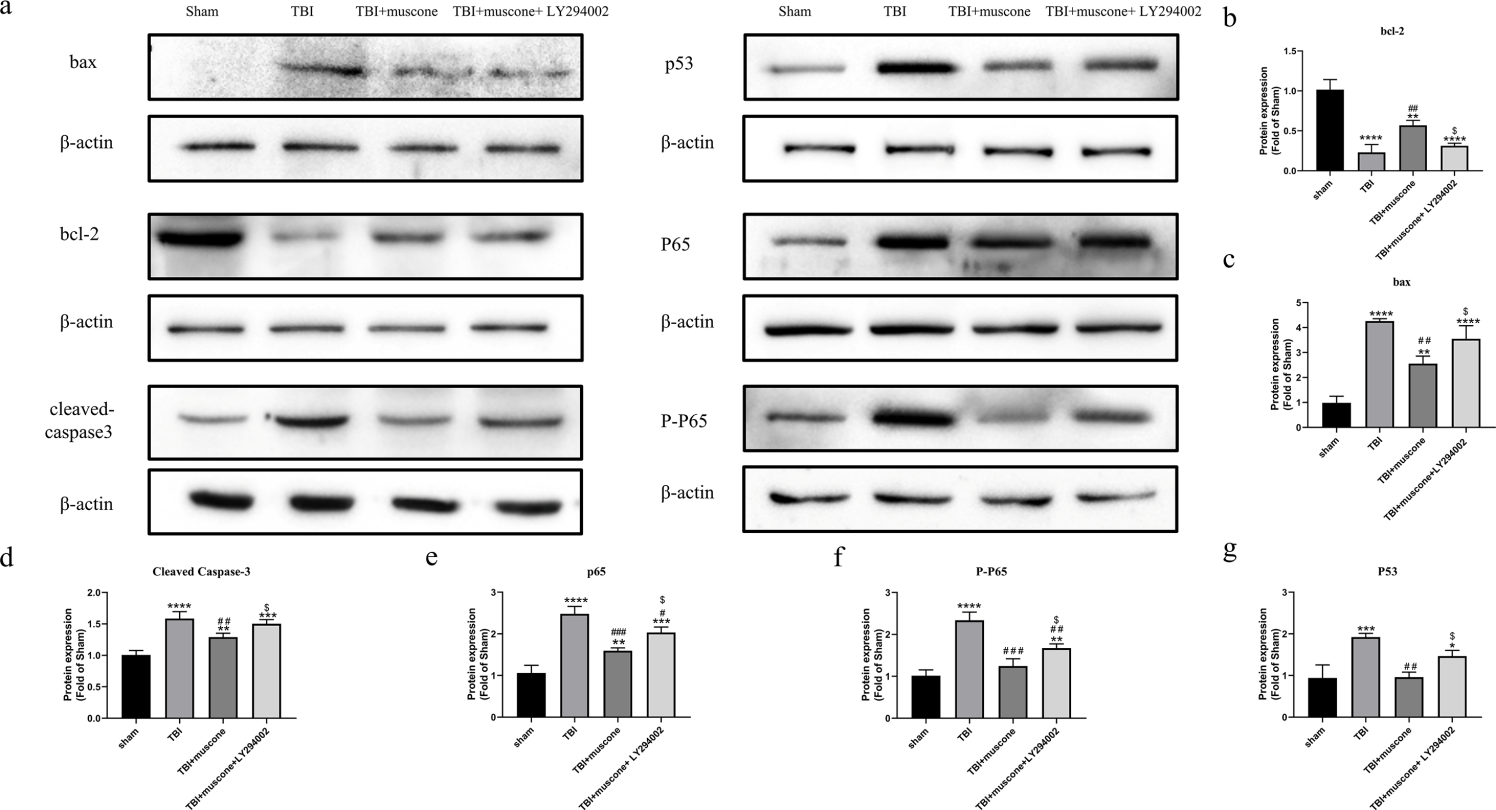
Effects of muscone on the bax, p53, BCL-2 and cleaved-caspase 3 expression and the NF-kB pathway. (A) Immunoblot bands in involved groups were represented. (B–G) The bands’ intensity was shown as optical density analysis. Results were presented as mean ± SD. *N* = 3, each group, ^∗^*P* < 0.05, ^∗∗^*P* < 0.01, ^∗∗∗^*P* < 0.001, ^∗∗∗∗^*P* < 0.0001 vs sham group; ^#^*p* < 0.05, ^##^*p* *lt*0.01, ^###^*p* < 0.001 vs TBI group; *p* < 0.05 vs TBI+muscone group.

LY294002 was used to block the Akt1 activity involved in regulating NF-kb in order to investigate the underlying molecular mechanism. According to quantitative results, both the phosphorylated NF-kB/p65 (p-p65) and NF-kB/p65 were significantly inhibited in the muscone-treated groups compared to those in the TBI group. We observed lower p-65 and p65 protein levels in the TBI + muscone + LY294002 group. The results indicated that LY294002 probably increased phosphorylated NF-kB/p65 and NF-kB/p65 when compared with the muscone + TBI groups through the PI3K/Akt1 pathway (Figs. 7A, 7E and 7F) (*P* < 0.05).

### Muscone reduced p53 expression 168 h after TBI

P53 affects the NF-kB pathway and acts as a key node to down-regulate neuroapoptosis (Li et al., 2018). Our results showed that the apoptosis-related levels (BCL-2, bax and cleaved caspase 3) were significantly altered 168 h after TBI compared to the sham group. Moreover, expression of these proteins were improved by muscone and offset by LY294002 (Figs. 7A–7D).

To further examine the effects of muscone on apoptosis, we conducted a Western blot analysis of the p53 expression. Quantitative analysis determined that there was a significant change between the TBI and sham groups. The p53 levels were significantly inhibited upon the injection of muscone compared to the TBI group. After TBI, higher levels of p53 were found in rats that were injected with LY294002 + muscone,compared to those in the TBI + muscone group (Fig. 7A and 7G) (*P* < 0.05). This suggests that muscone may ameliorate p53-mediated apoptosis through the PI3K/Akt pathway.

## Discussion

Natural medicine is now receiving greater attention for its multiple active ingredients and potential targets. Numerous medicinal plants containing XNJI have been used clinically to treat TBI in China. However, the bioactive components of XNJI and its mechanisms for treating TBI are unknown. It is imperative to investigate the active components in XNJI for clinical use.

Previous research has illustrated XNJI’s neuroprotective properties (Qu et al., 2019; Wei et al., 2015; Zhang et al., 2018), but there has been no network pharmacology analysis to investigate the combination of TBI and XNJI. We constructed a systems pharmacological model for XNJI by screening active ingredients, identifying targets, analyzing PPI, conducting enrichment analysis of KEGG and GO, and verifying experiments. We identified 35 bioactive ingredients in XNJI and 173 potential targets using network pharmacology. The active ingredients work with different target proteins and may outperform single-target ingredients. PPI, GO, and KEGG analysis were typically involved in apoptosis, PI3K-Akt, p53, and the cell cycle signaling pathway. XNJI and muscone had a positive effect on apoptosis-related mRNA 168 h after TBI. Through network analysis, muscone was found to have close relationships with many targets. Muscone may reduce brain damage by preventing the pro-apoptotic proteins bax and caspase 3 (Wei et al., 2012), which was consistent with the XNJI results (Zhang et al., 2018). Muscone was further studied and found to perform a similar function in inhibiting apoptosis in TBI. This effect may be partially offset by LY294002.

Akt1, RELA, CASP3 were each enriched in the PI3K/Akt, NF-kB, and apoptosis signaling pathways. The p53 pathway played a greater role in the hub genes-related pathways. The p53 tumor suppressor gene regulated DNA-damage-induced apoptosis (Crighton et al., 2006; Sánchez et al., 2014) and the p53 expression level was down-regulated by XNJI and muscone. Previous evidence suggested that XNJI could protect brain cells against damage by inhibiting the expression of p53 (Wei et al., 2015). Our results demonstrated that muscone decreased apoptosis by inhibiting the NF-kB and signaling pathways of PI3K/Akt1. When PI3K/Akt1 was reduced by LY294002, NF-kB also had the same effect. Interestingly, the caspase-dependent pathways were often inhibited by up-regulating NF-kB and Akt in some diseases (Gutjahr et al., 2020; Shamekhi et al., 2020). This may result from inflammation in the PI3K/Akt and NF-kB pathways. Multiple studies have shown that inflammation factors are elevated in neuroinflammation through the PI3K/Akt and NF-kB pathways during brain injury (Amin, Shah & Kim, 2016; Zhu et al., 2018). NF-kB is the direct target of AKT and is vital for TNF-a expression (Amin, Shah & Kim, 2016; Fu et al., 2018). Research has indicated that the extrinsic apoptotic pathway can be triggered by inflammatory factors such as TNF and Fas (Akamatsu & Hanafy, 2020). Also,one study showed that inhibition of microglia may decrease neuroinflammation and neuronal apoptosis by altering their polarization status to reduce the M1 phenotype marker TNF-*α* after TBI (Takada et al., 2020). XNJI may also improve cerebral I/R injury and decreased the expression levels of inflammatory mediators involving TNF-a, IL-1,*β* and IL-6 (Zhang et al., 2020). Muscone has been shown to be neuroprotective in stroke by reducing the Fas level (Wei et al., 2012). Therefore, it is possible that muscone primarily acts on extrinsic apoptotic activation, relying on the inhibition of inflammatory pathways. However, Li et al. (2018) found that repressing p53 activation attenuated neuroinflammation and neuroapoptosis via the downregulation of caspase 3 and the NF-kB cytokine pathway. There was dual crosstalk between p53 and NF-kB (p65, RELA) which leads them to antagonize or activate each other (Schneider & Krämer, 2011). Intriguingly, muscone was used in conjunction with LY294002(PI3K inhibitor), while PI3K/Akt1 pathway was increased compared to muscone alone. Possibly, this is because LY294002 caused a decrease for muscone in blood–brain barrier permeability or the combination of muscone and LY294002 abolished muscone’s effect on TBI.

These effects should be included in future research on these underlying mechanisms. Our results demonstrated that muscone may relieve apoptosis by inhibiting the NF-kB pathway, PI3K-Akt1, and p53 signaling pathways through their interactions.

## Conclusion

In a nutshell, muscone is an effective ingredient in XNJI, which can prevent TBI and was revealed through network analysis. Moreover,muscone can ameliorate apoptosis–related proteins which is parallel to XNJI. It may function via NF-kB, p53 and PI3K/Akt1 signaling pathways. Future studies should focus on identifying lead compounds in the natural products of pharmacological systems. There are many active chemical components in XNJI but their molecular mechanisms are still unclear and the technology for analyzing XNJI is flawed. Additional research is still needed.

## Supplemental Information

10.7717/peerj.11696/supp-1 Supplemental Information 1 Gels/Blots Full-length uncropped blots

10.7717/peerj.11696/supp-2 Supplemental Information 2 Schematic representations of drug administration after TBI (upper)XNJI or muscone treatment at 24 h,72 h and 168h respectively after TBI in rats.(lower)muscone , muscone+ LY294002 treatment and TBI exposure at 168 h after TBI.

10.7717/peerj.11696/supp-3 Supplemental Information 3 Effects of muscone on the bax, BCL-2 and caspase-3 activation and the PI3K/Akt pathway (A and E) Representative images of immunohistochemical staining for bax, BCL-2 , caspase-3,PI3K and Akt(scale bar = 100 um). (B–D, F and G) Quantification of immunohistochemistry results. *N* = 3,each group ^∗^*P* < 0.05, ^∗∗^*P* < 0.01, ^∗∗∗^*P* < 0.001, ^∗∗∗∗^*P* < 0.0001 vs sham group; ^#^*p* < 0.05, ^##^*p* < 0.01, ^###^*p* < 0.001, ^###^*p* < 0.0001 vs TBI group; *p* < 0.05 vs TBI+muscone group.

10.7717/peerj.11696/supp-4 Supplemental Information 4 The virtual docking methods and situation of muscone and Akt1

10.7717/peerj.11696/supp-5 Supplemental Information 5 The ARRIVE guidelines 2.0: author checklist

10.7717/peerj.11696/supp-6 Supplemental Information 6 The detailed information of Venn, network, GO, KEGG and PPI diagram

## Additional Information and Declarations

### Data Availability

The following information was supplied regarding data availability: The raw measurements are available in the Supplemental Files.
