# Supplementary figures and images for: Network pharmacology to investigate the pharmacological mechanisms of muscone in Xingnaojing injections for the treatment of severe traumatic brain injury
Source: PeerJ. 2021 Jul 20;9:e11696. doi: 10.7717/peerj.11696 (PMC8300495; doi:10.7717/peerj.11696)

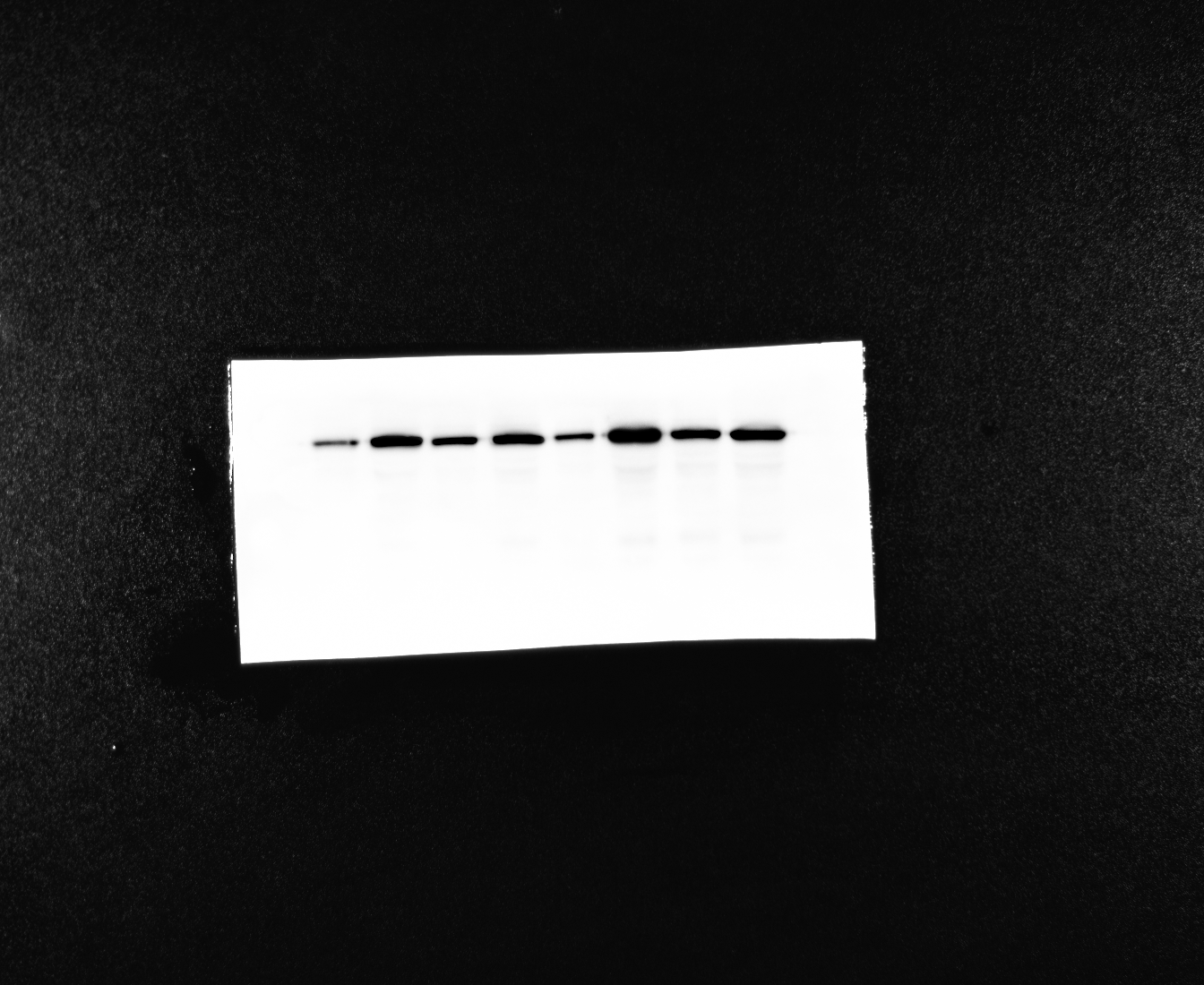

Supplement: Supplemental Information 1 — Full-length uncropped blots [file peerj-09-11696-s001.zip › WB/akt.tif]

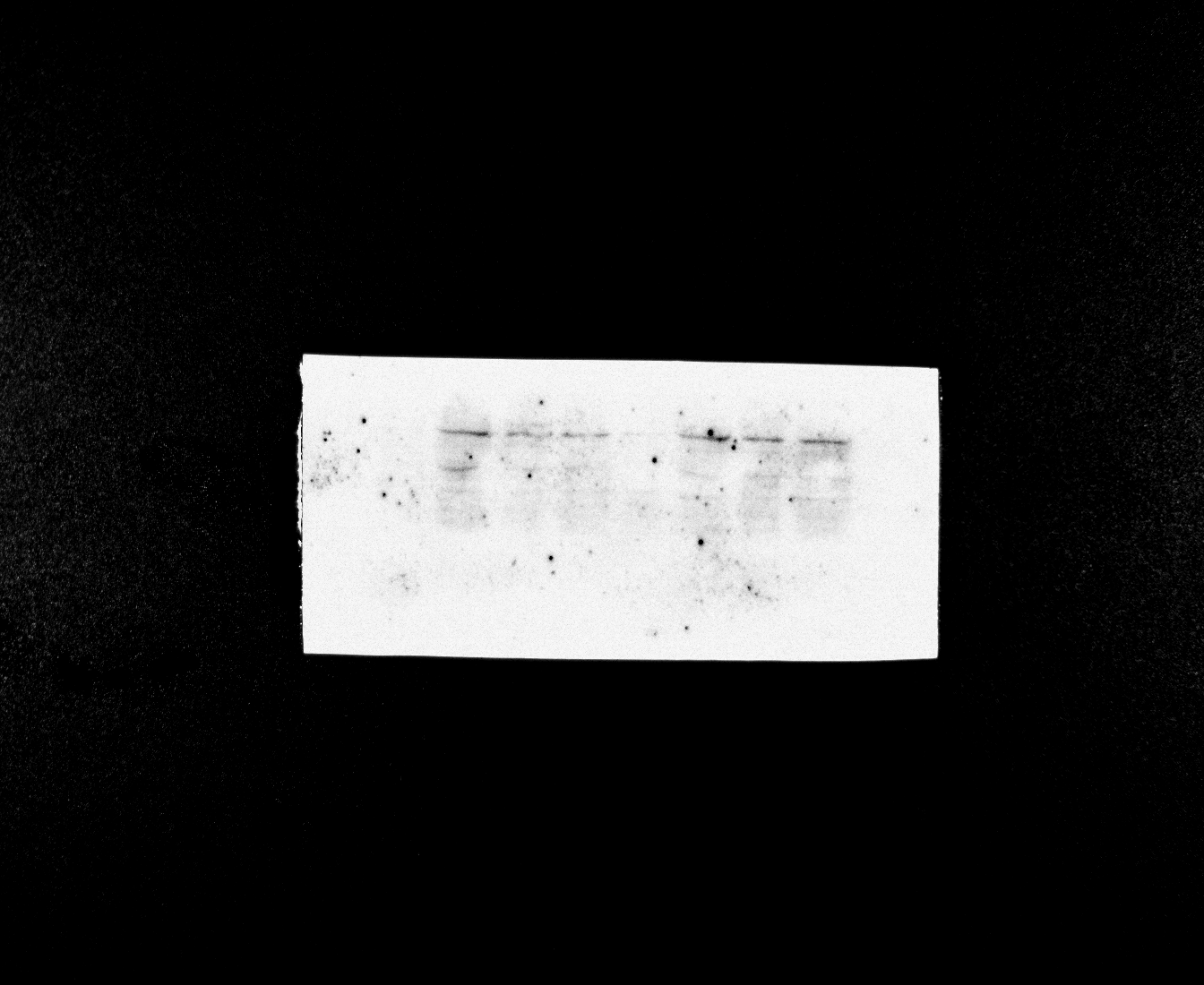

Supplement: Supplemental Information 1 — Full-length uncropped blots [file peerj-09-11696-s001.zip › WB/BAX.tif]

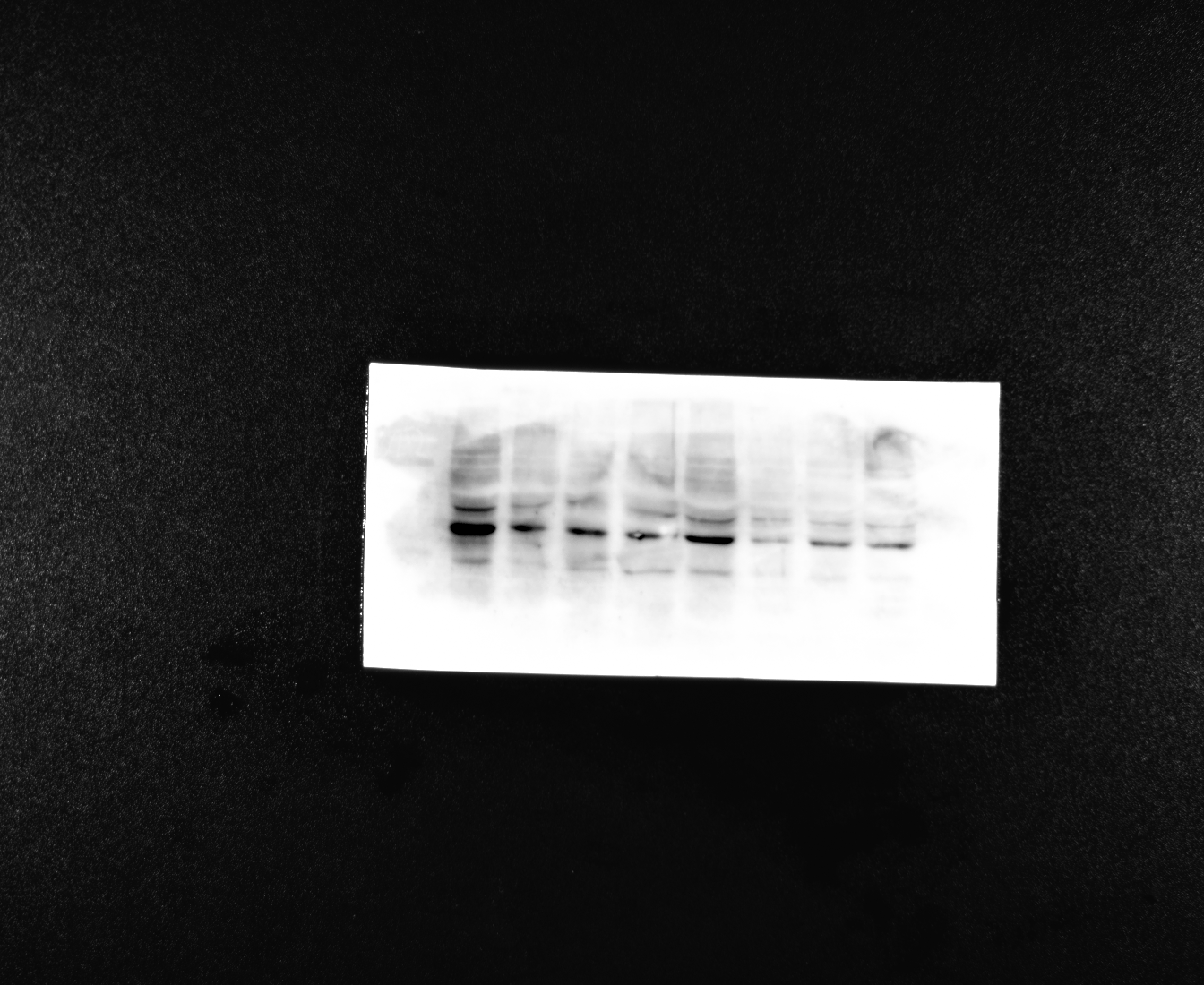

Supplement: Supplemental Information 1 — Full-length uncropped blots [file peerj-09-11696-s001.zip › WB/Bcl-2.tif]

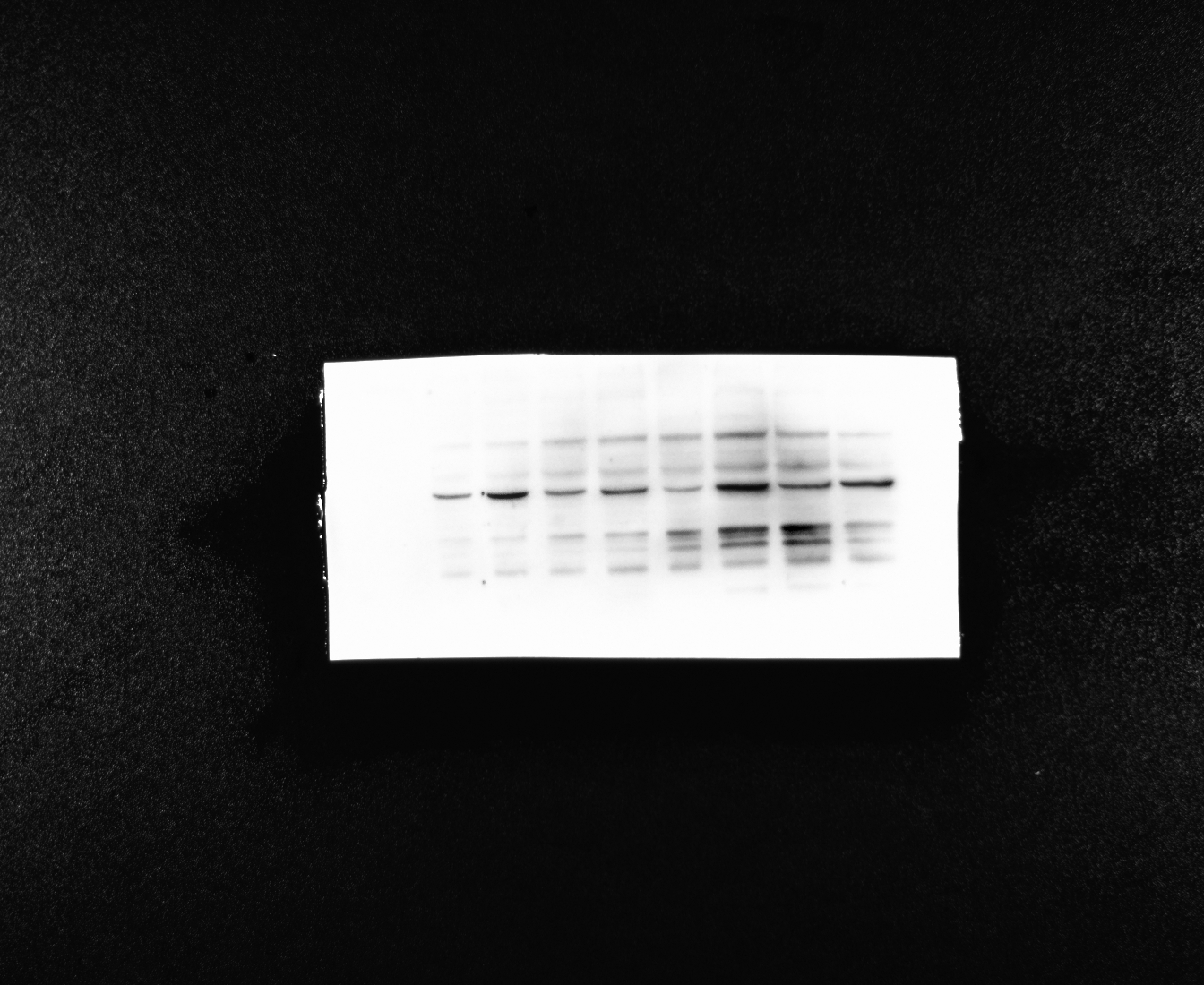

Supplement: Supplemental Information 1 — Full-length uncropped blots [file peerj-09-11696-s001.zip › WB/cleaved-cp3.tif]

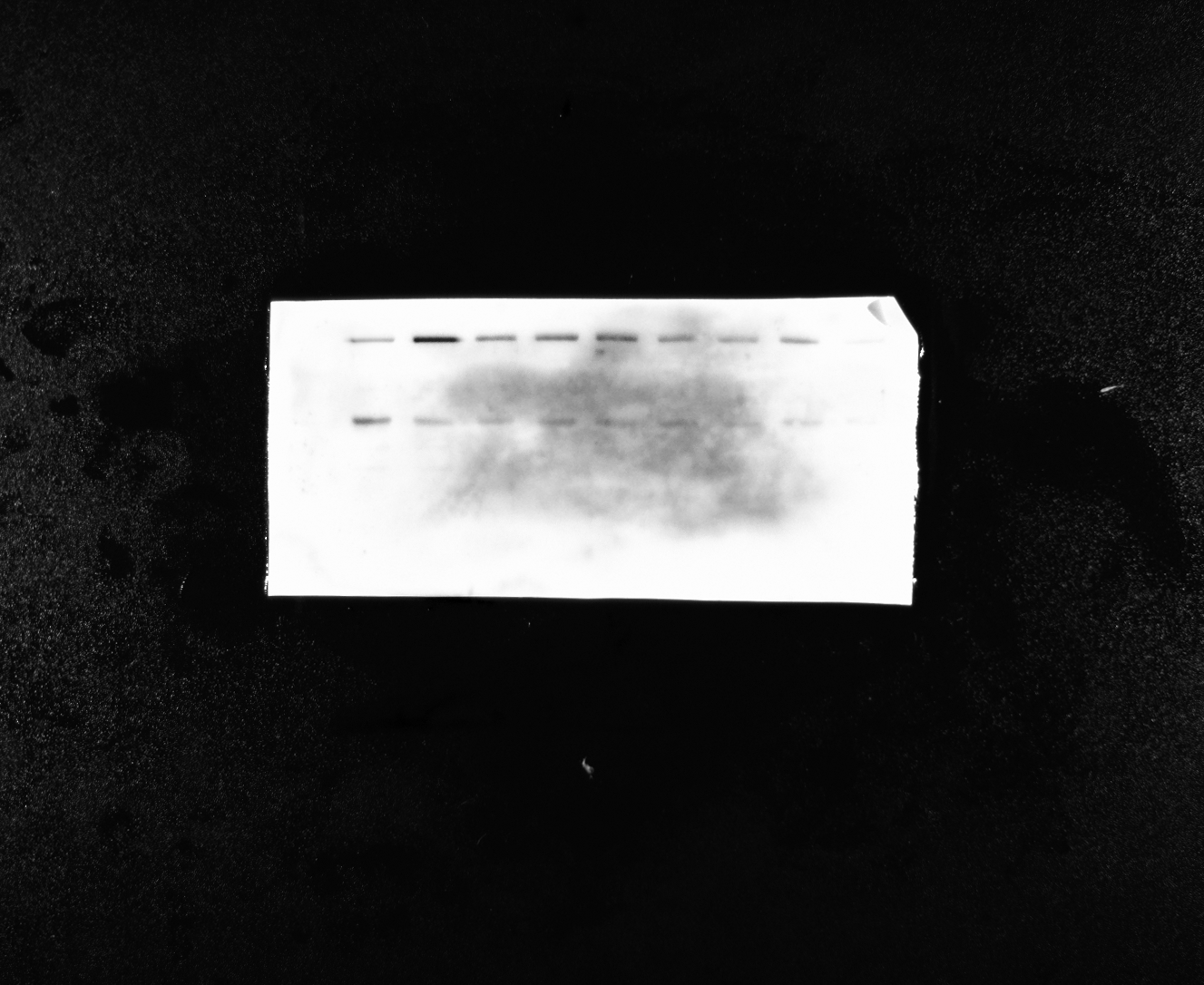

Supplement: Supplemental Information 1 — Full-length uncropped blots [file peerj-09-11696-s001.zip › WB/p-53.tif]

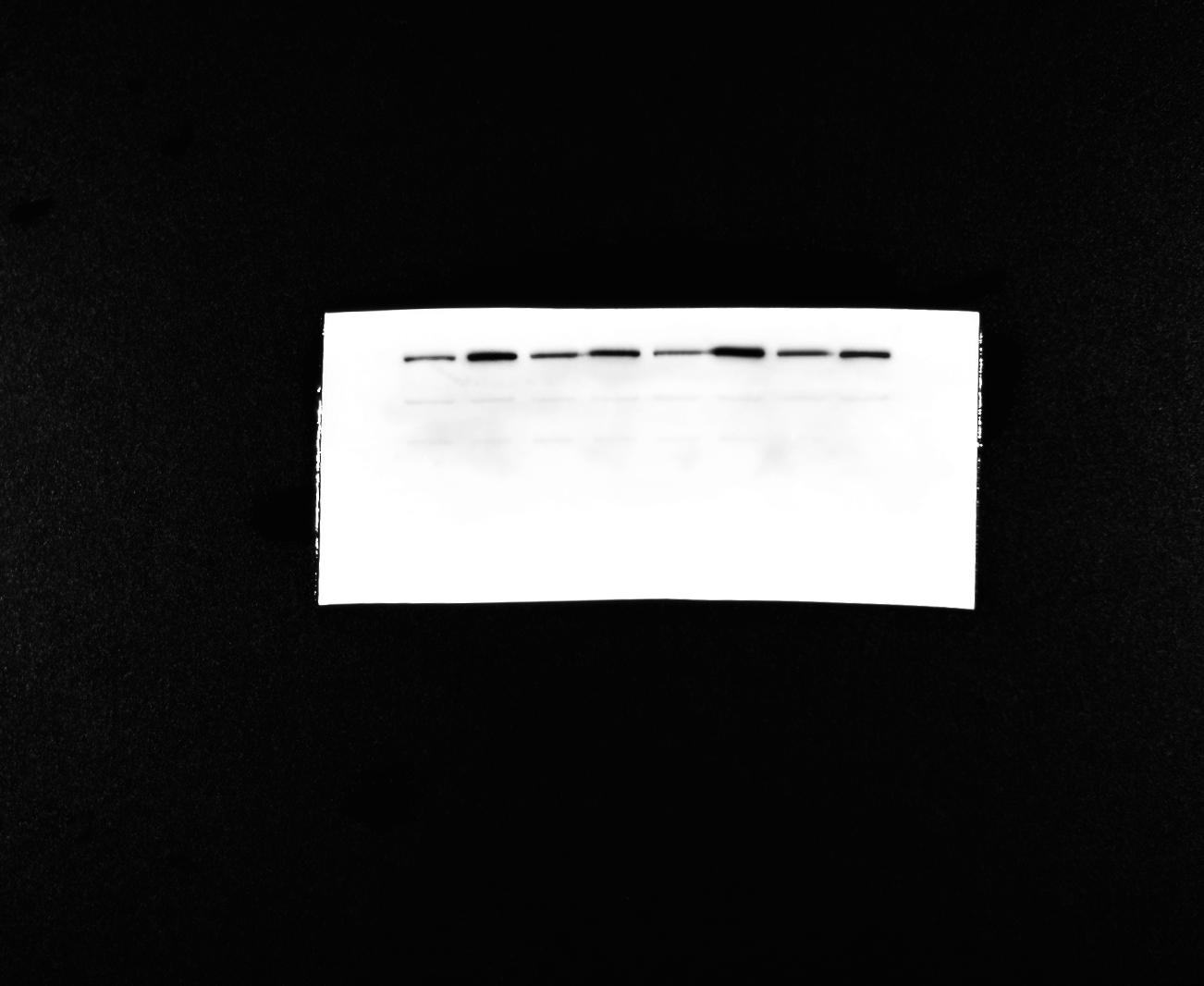

Supplement: Supplemental Information 1 — Full-length uncropped blots [file peerj-09-11696-s001.zip › WB/p-AKT.tif]

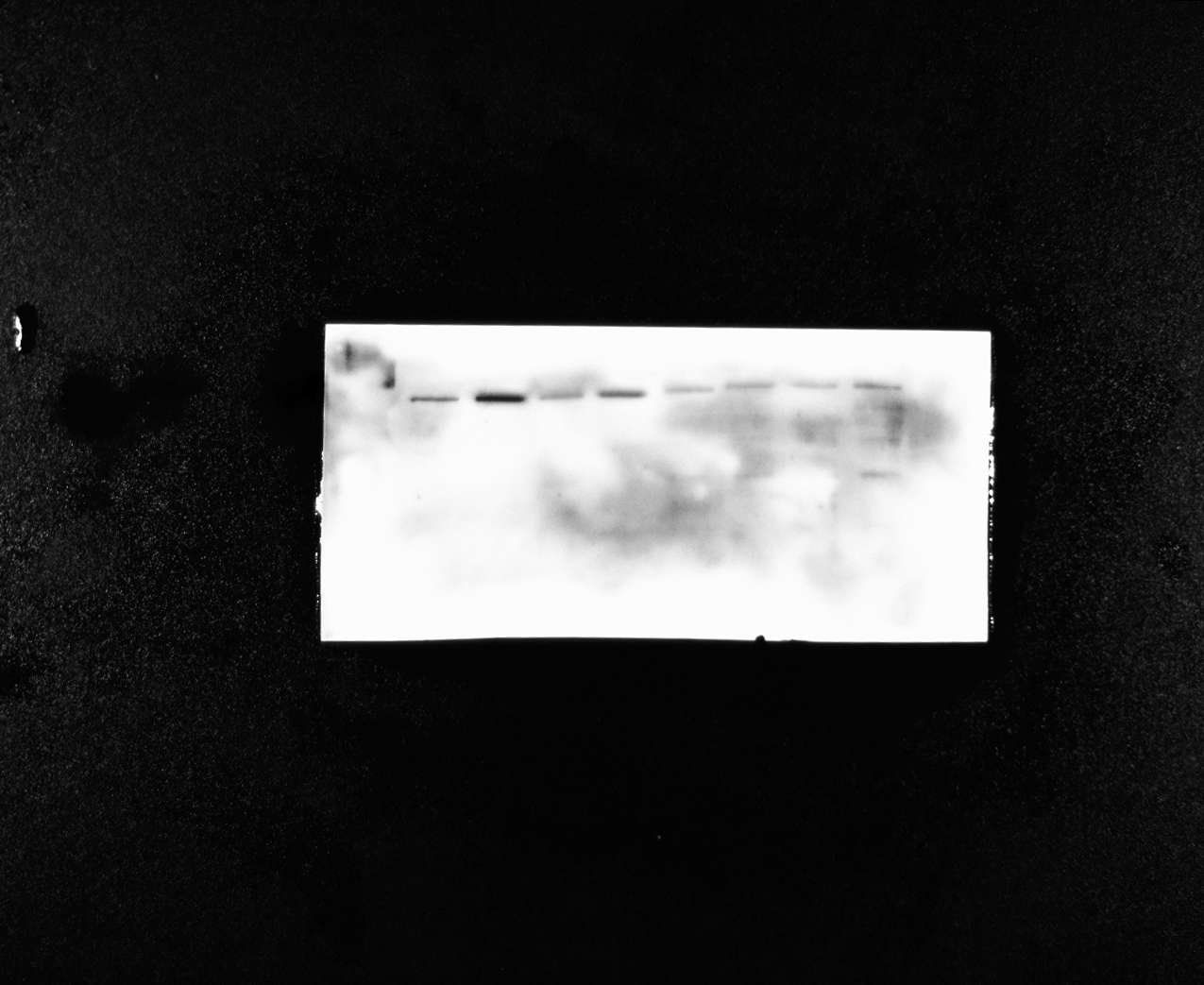

Supplement: Supplemental Information 1 — Full-length uncropped blots [file peerj-09-11696-s001.zip › WB/p-p65.tif]

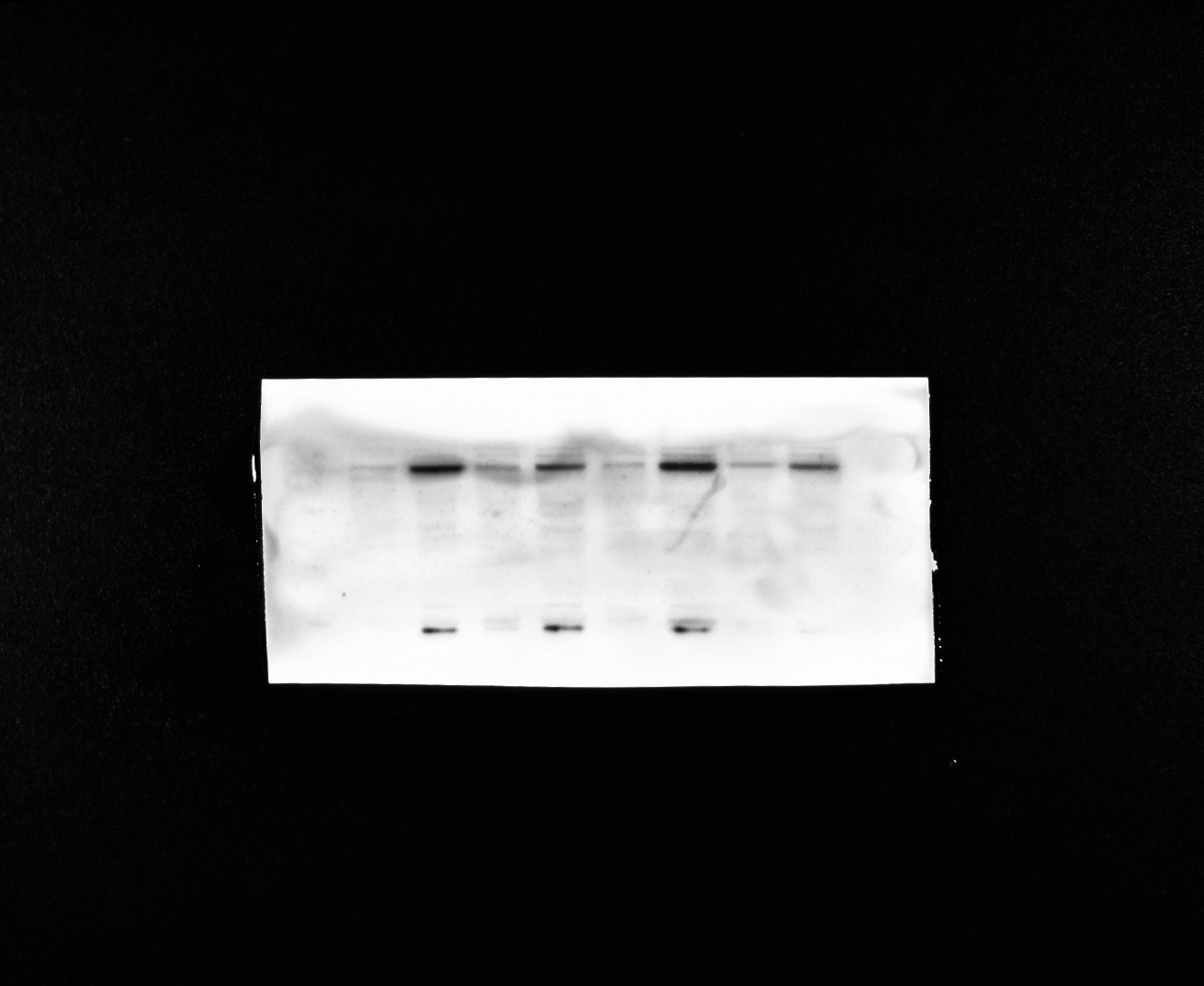

Supplement: Supplemental Information 1 — Full-length uncropped blots [file peerj-09-11696-s001.zip › WB/p-pi3k.tif]

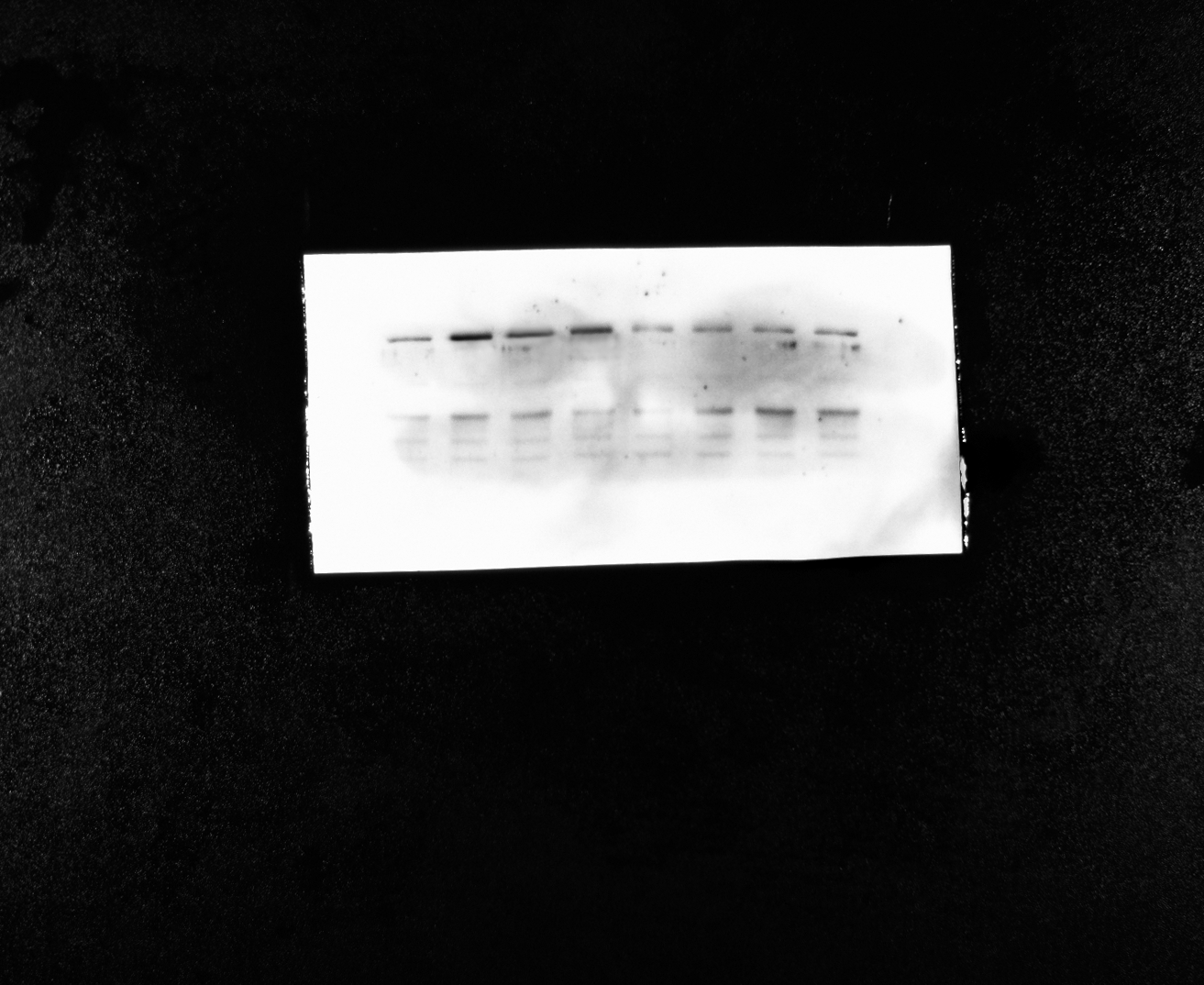

Supplement: Supplemental Information 1 — Full-length uncropped blots [file peerj-09-11696-s001.zip › WB/p65.tif]

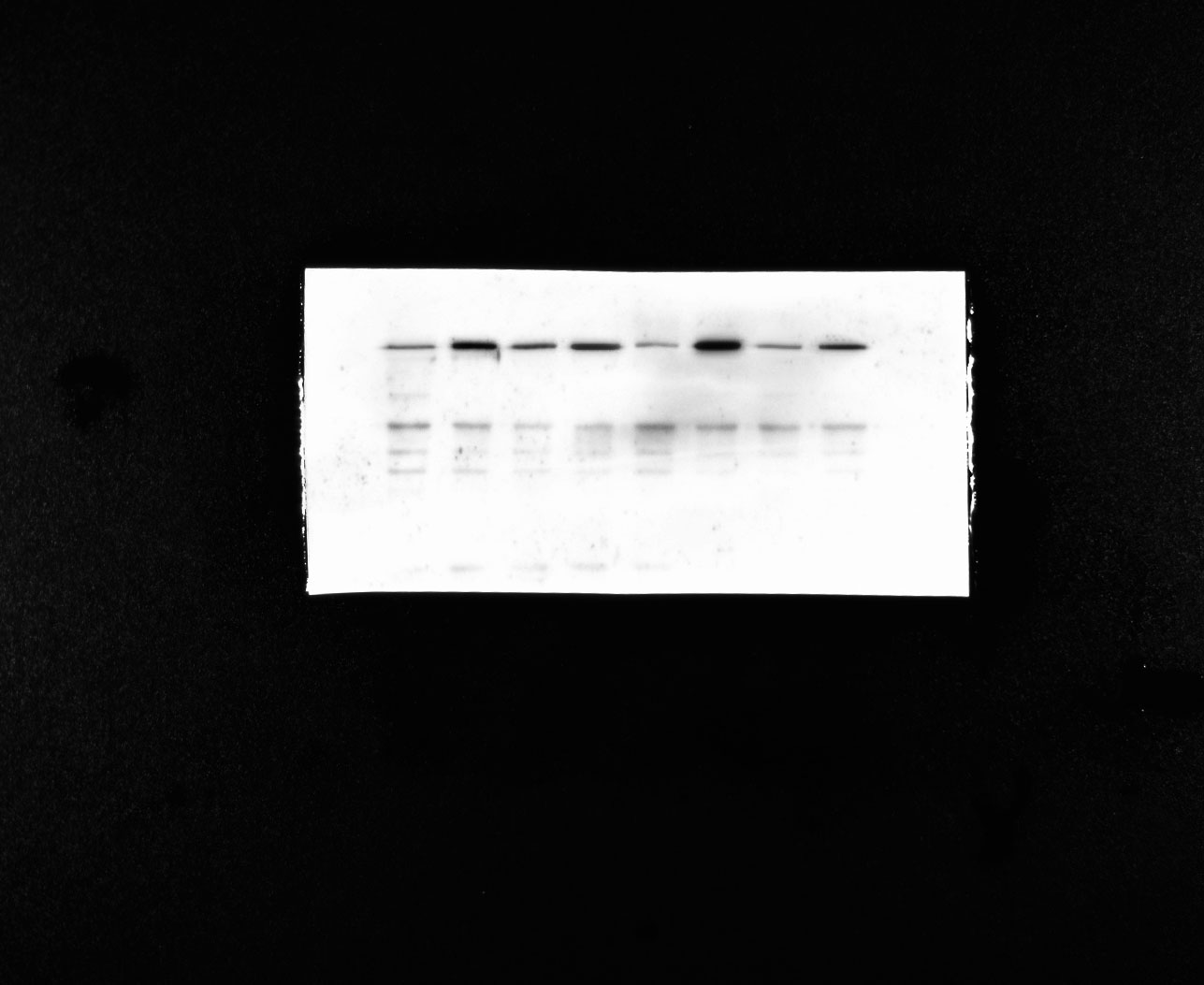

Supplement: Supplemental Information 1 — Full-length uncropped blots [file peerj-09-11696-s001.zip › WB/PI3K.tif]

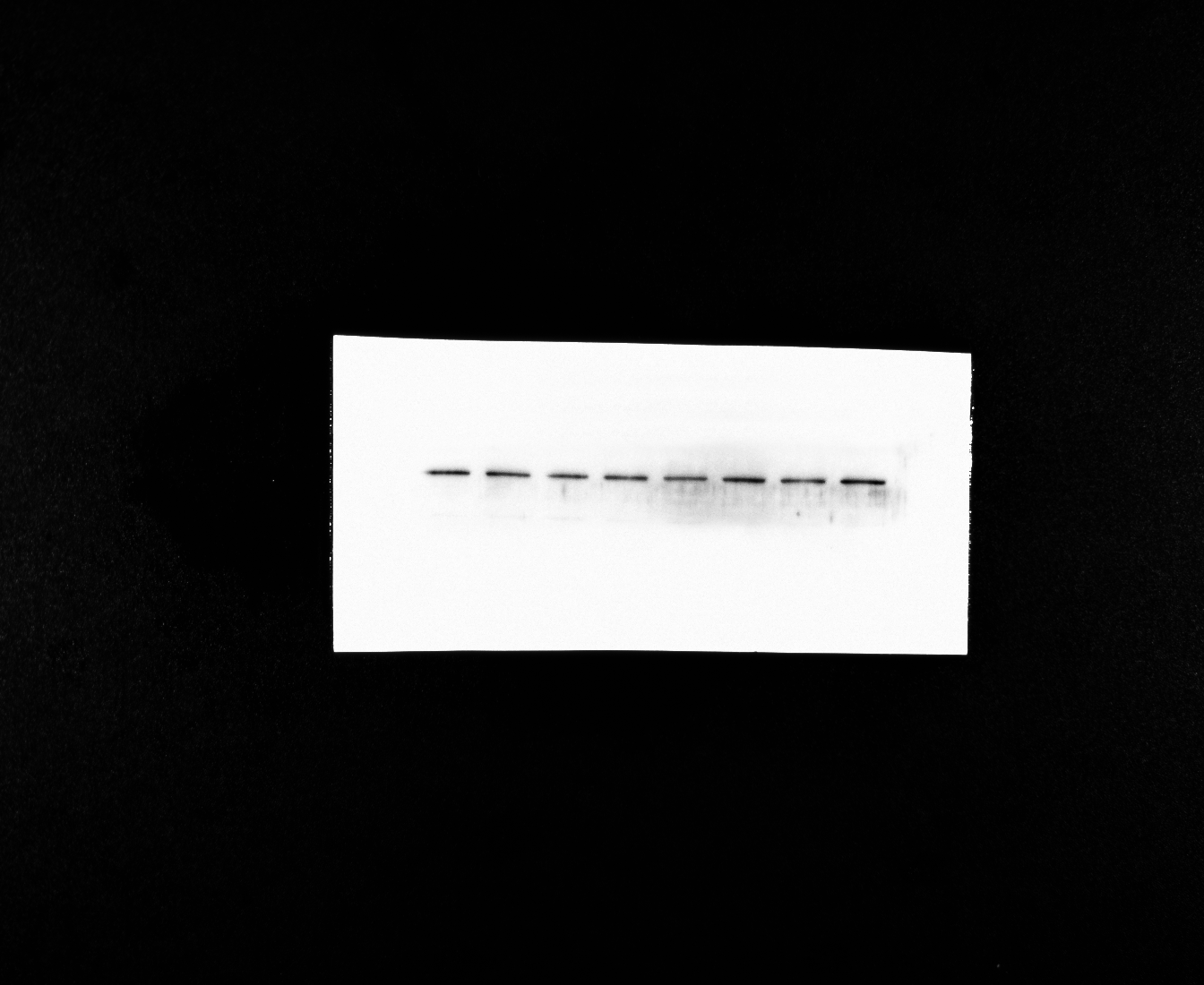

Supplement: Supplemental Information 1 — Full-length uncropped blots [file peerj-09-11696-s001.zip › WB/内1.tif]

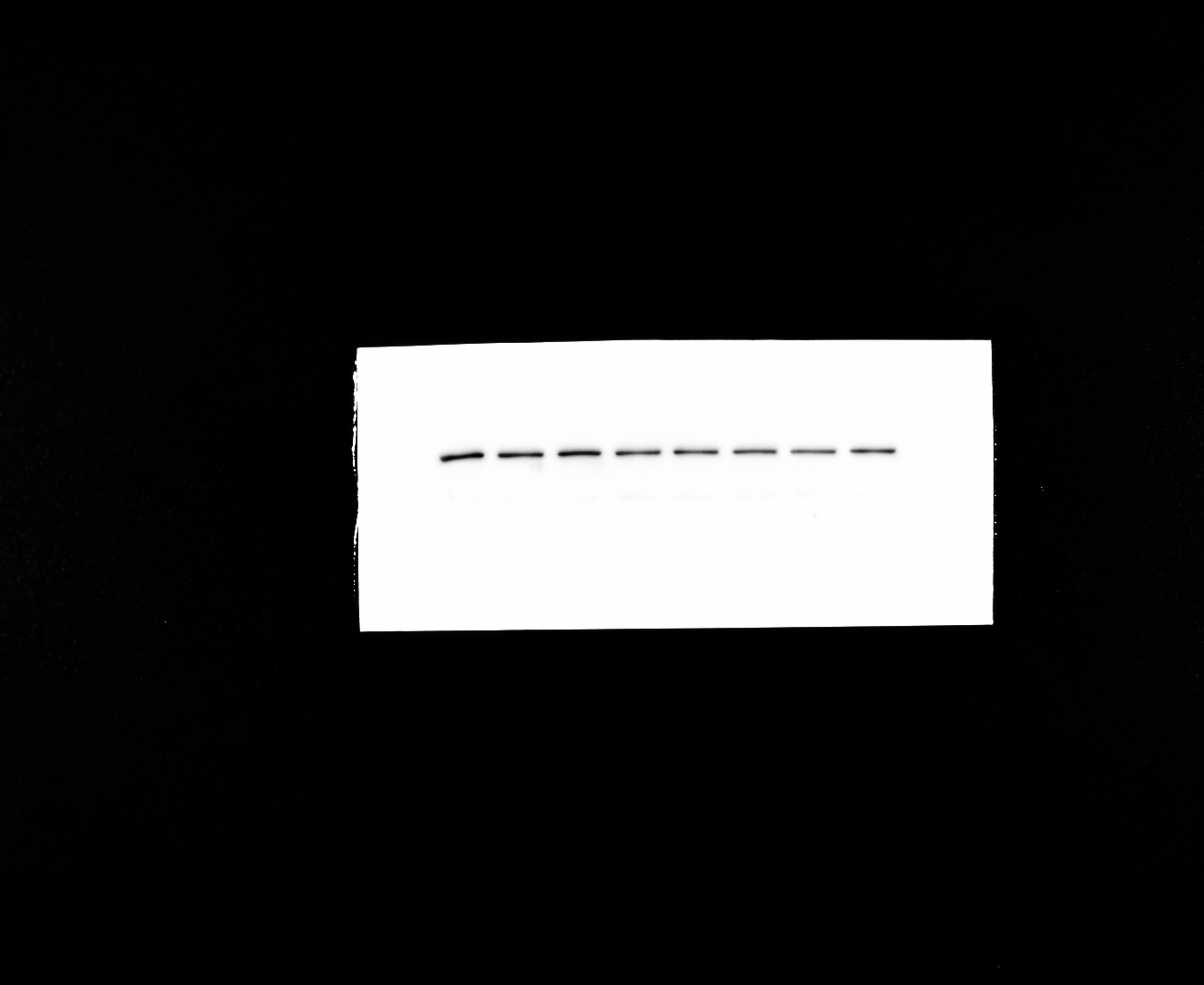

Supplement: Supplemental Information 1 — Full-length uncropped blots [file peerj-09-11696-s001.zip › WB/内2.tif]

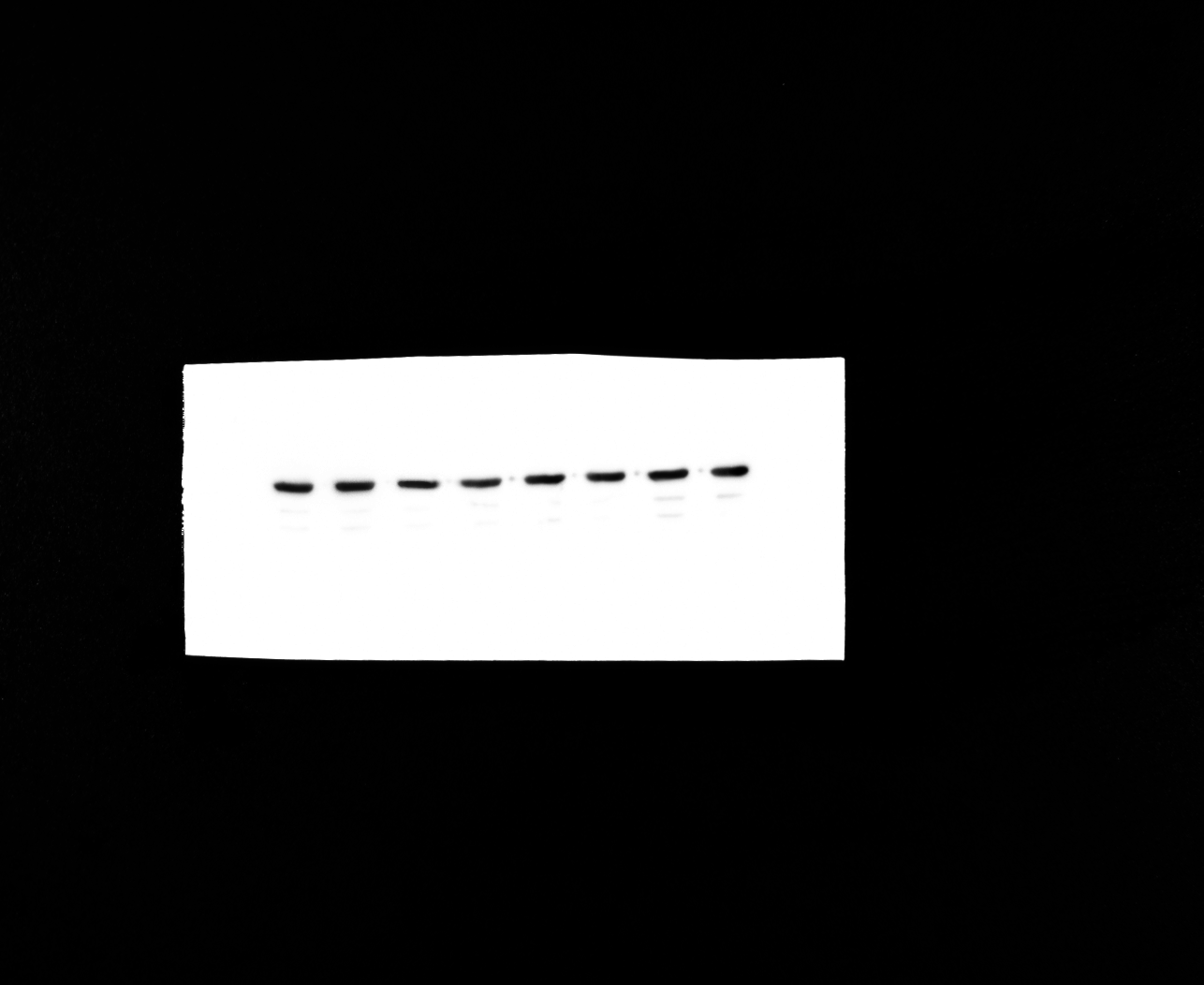

Supplement: Supplemental Information 1 — Full-length uncropped blots [file peerj-09-11696-s001.zip › WB/内3.tif]

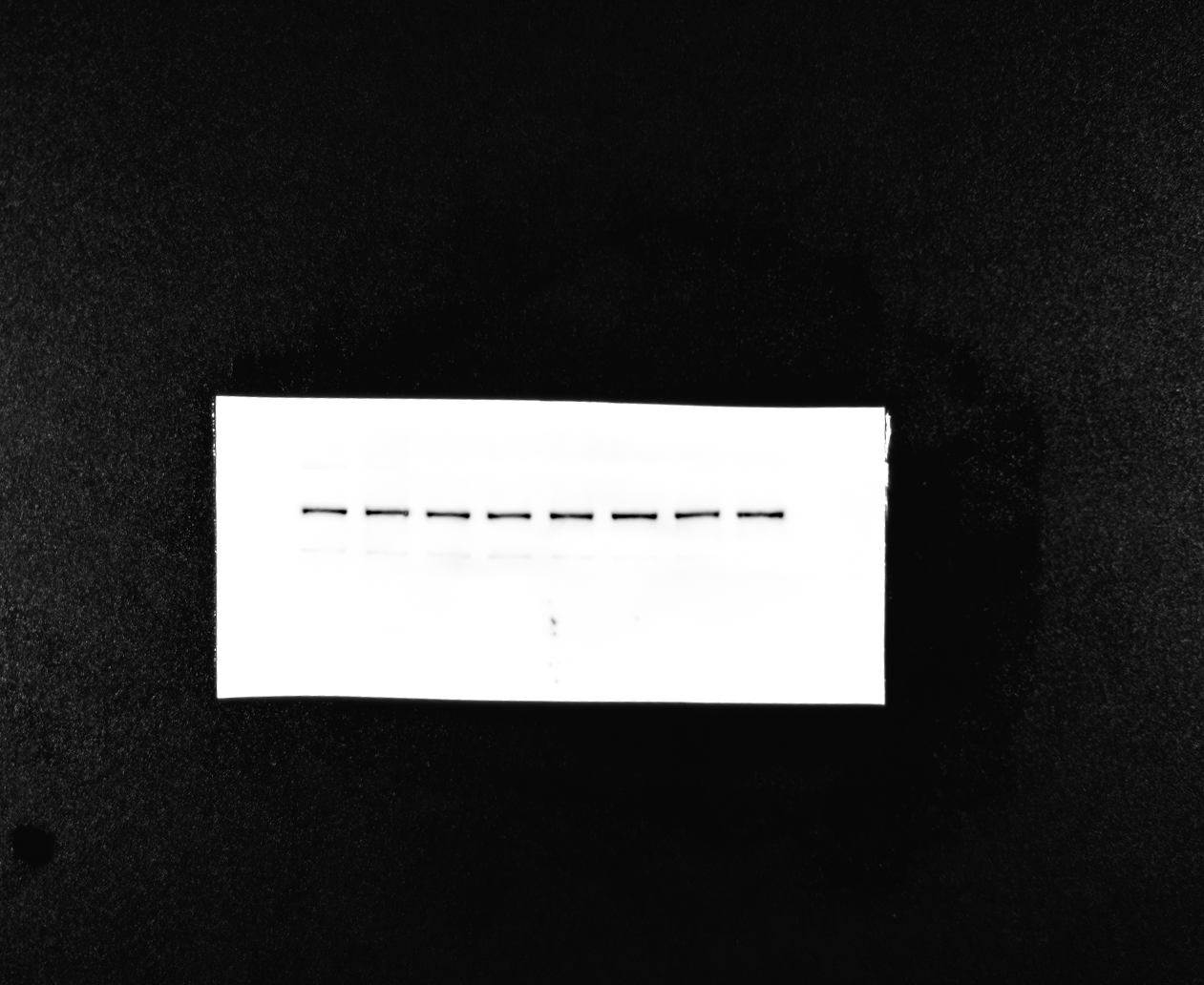

Supplement: Supplemental Information 1 — Full-length uncropped blots [file peerj-09-11696-s001.zip › WB/内4.tif]

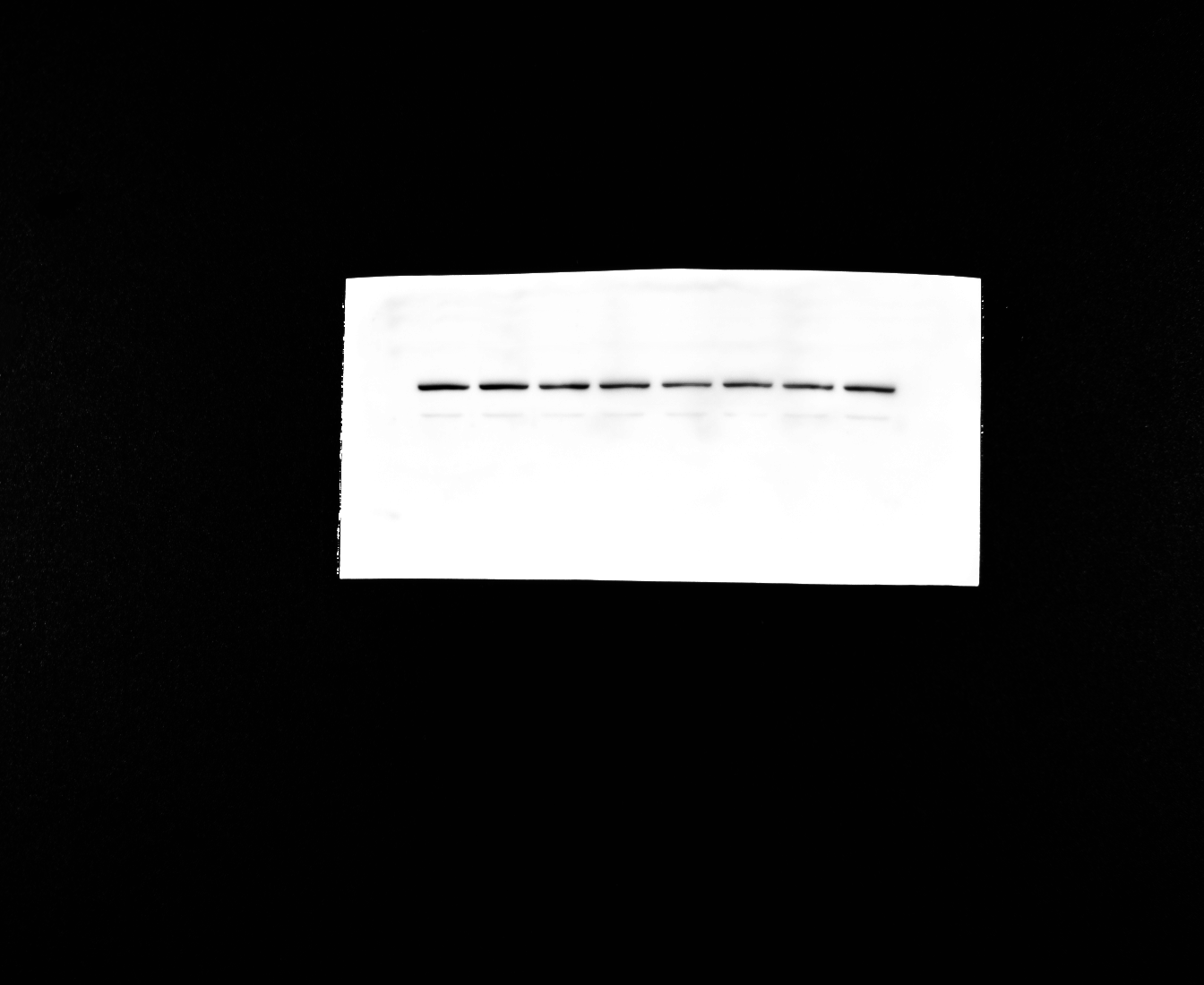

Supplement: Supplemental Information 1 — Full-length uncropped blots [file peerj-09-11696-s001.zip › WB/内5.tif]

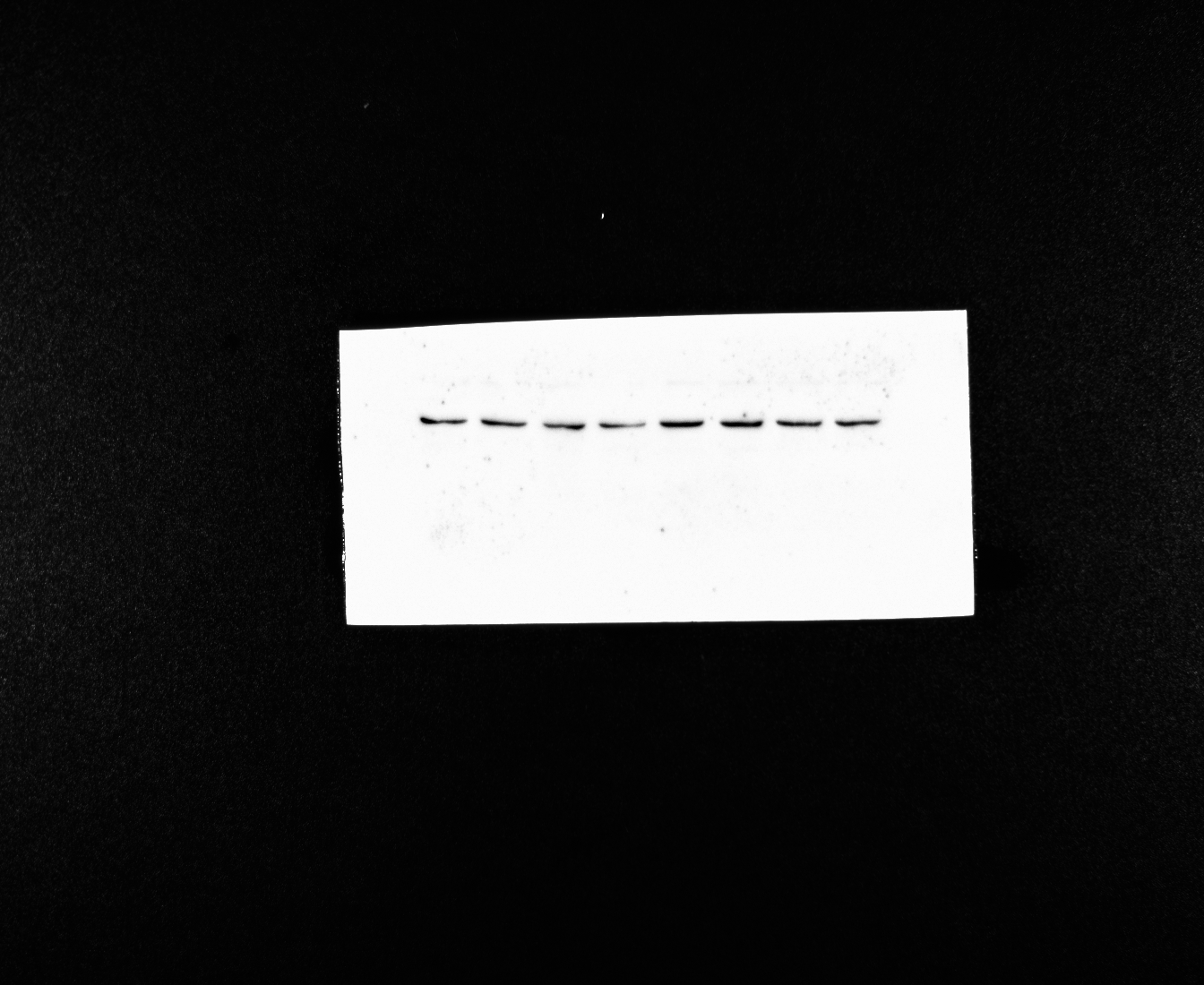

Supplement: Supplemental Information 1 — Full-length uncropped blots [file peerj-09-11696-s001.zip › WB/内6.tif]

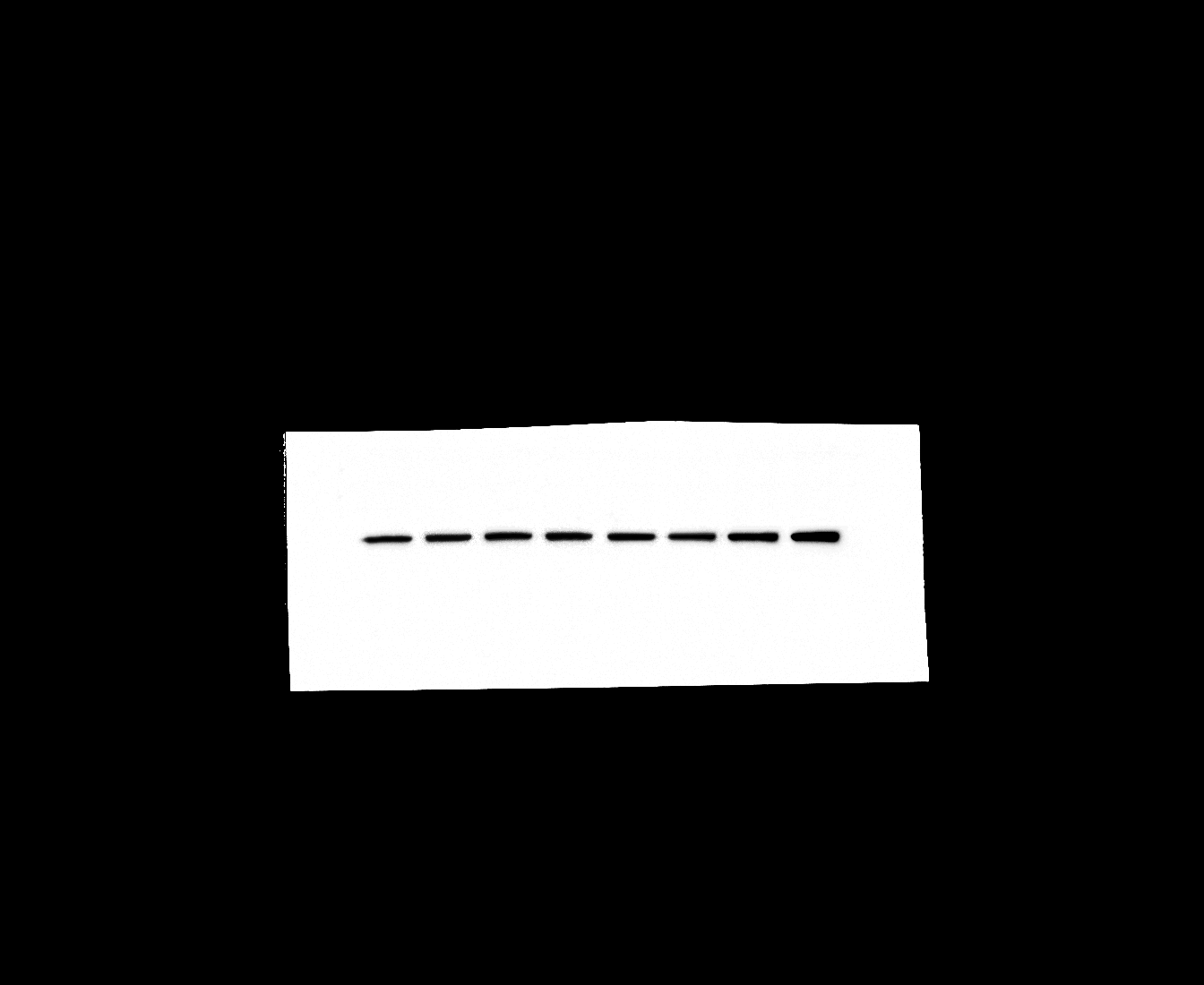

Supplement: Supplemental Information 1 — Full-length uncropped blots [file peerj-09-11696-s001.zip › WB/内7.tif]

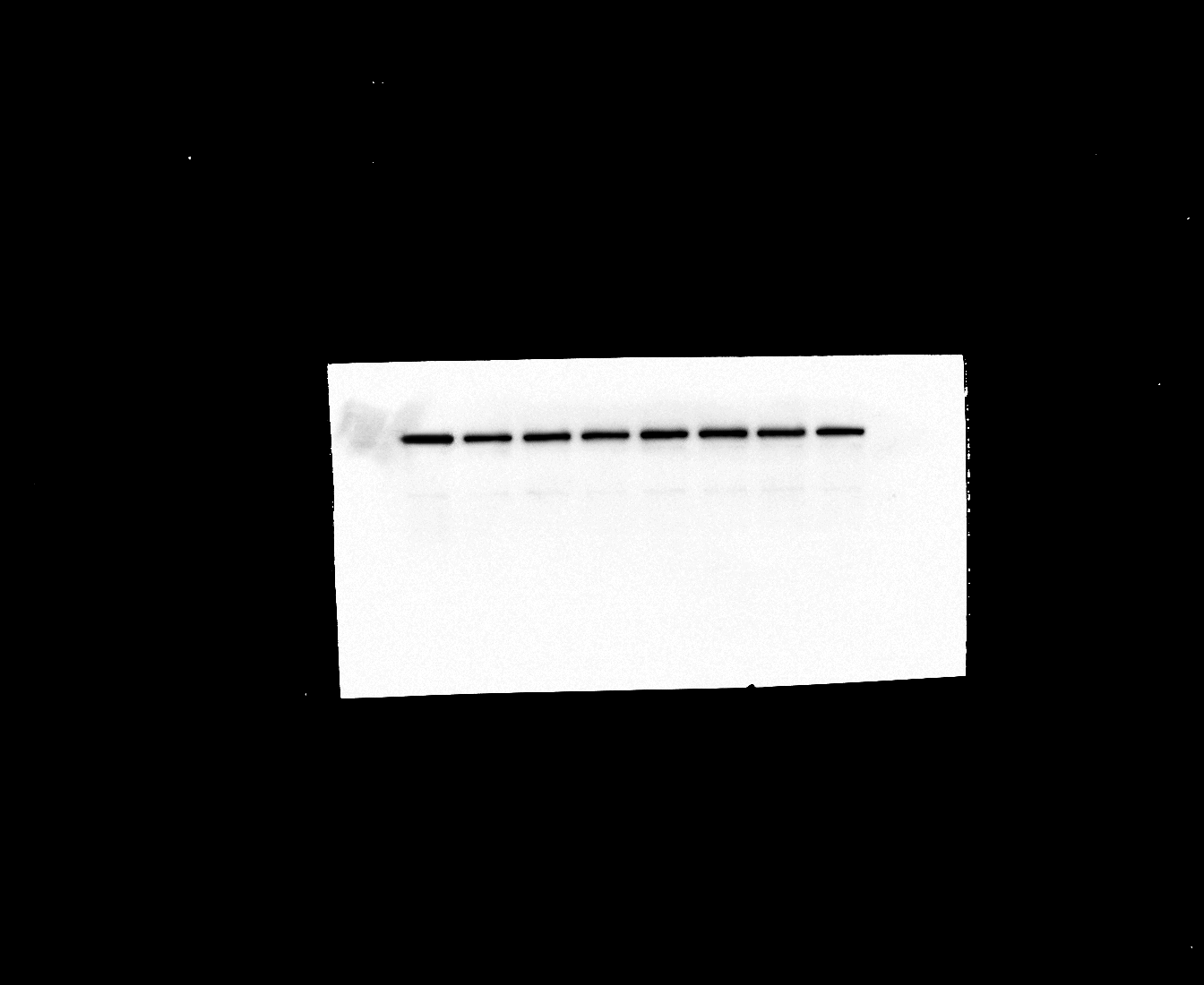

Supplement: Supplemental Information 1 — Full-length uncropped blots [file peerj-09-11696-s001.zip › WB/内8.tif]

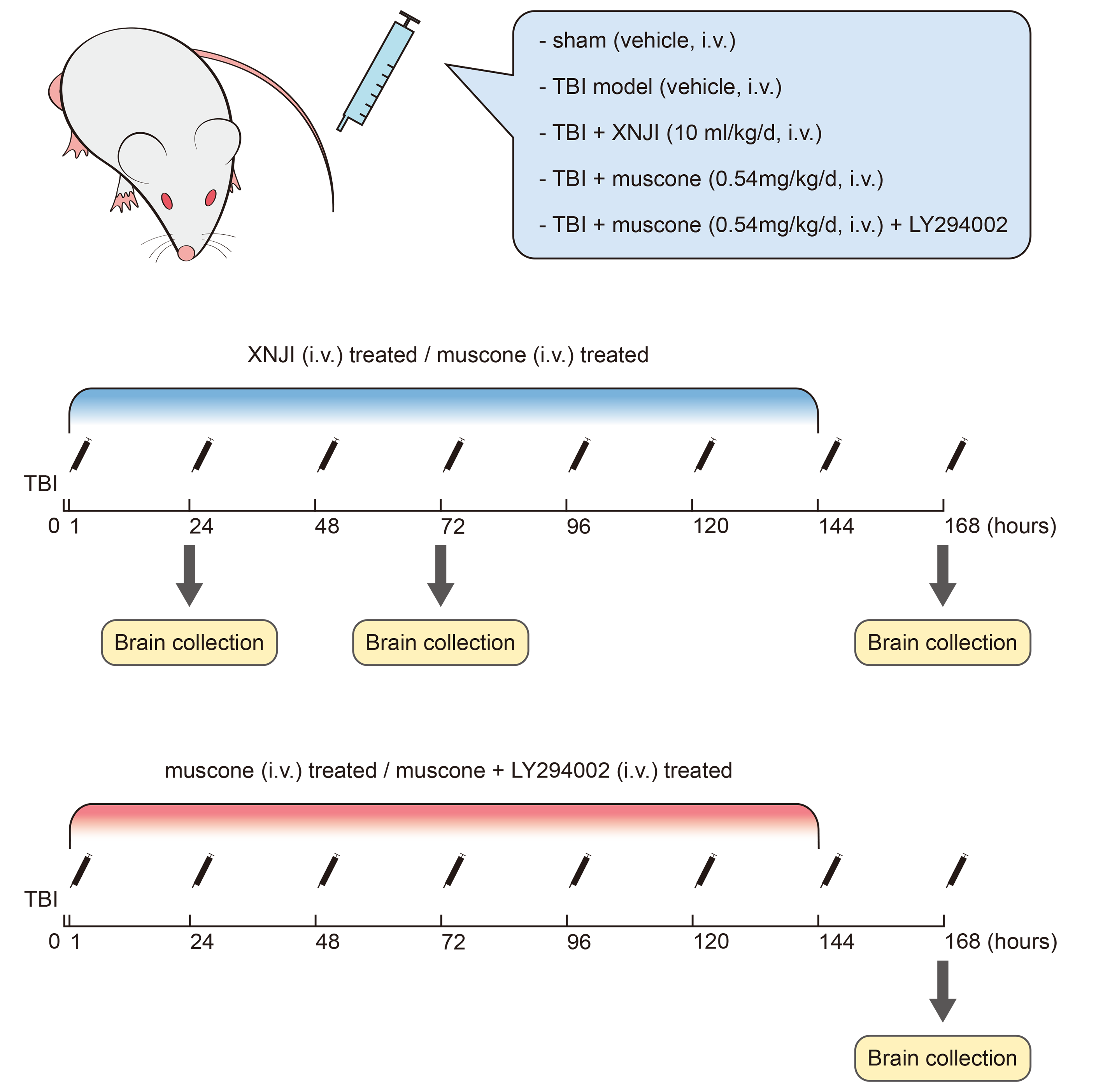

Supplement: Supplemental Information 2 — (upper)XNJI or muscone treatment at 24 h,72 h and 168h respectively after TBI in rats.(lower)muscone , muscone+ LY294002 treatment and TBI exposure at 168 h after TBI. [file peerj-09-11696-s002.png]

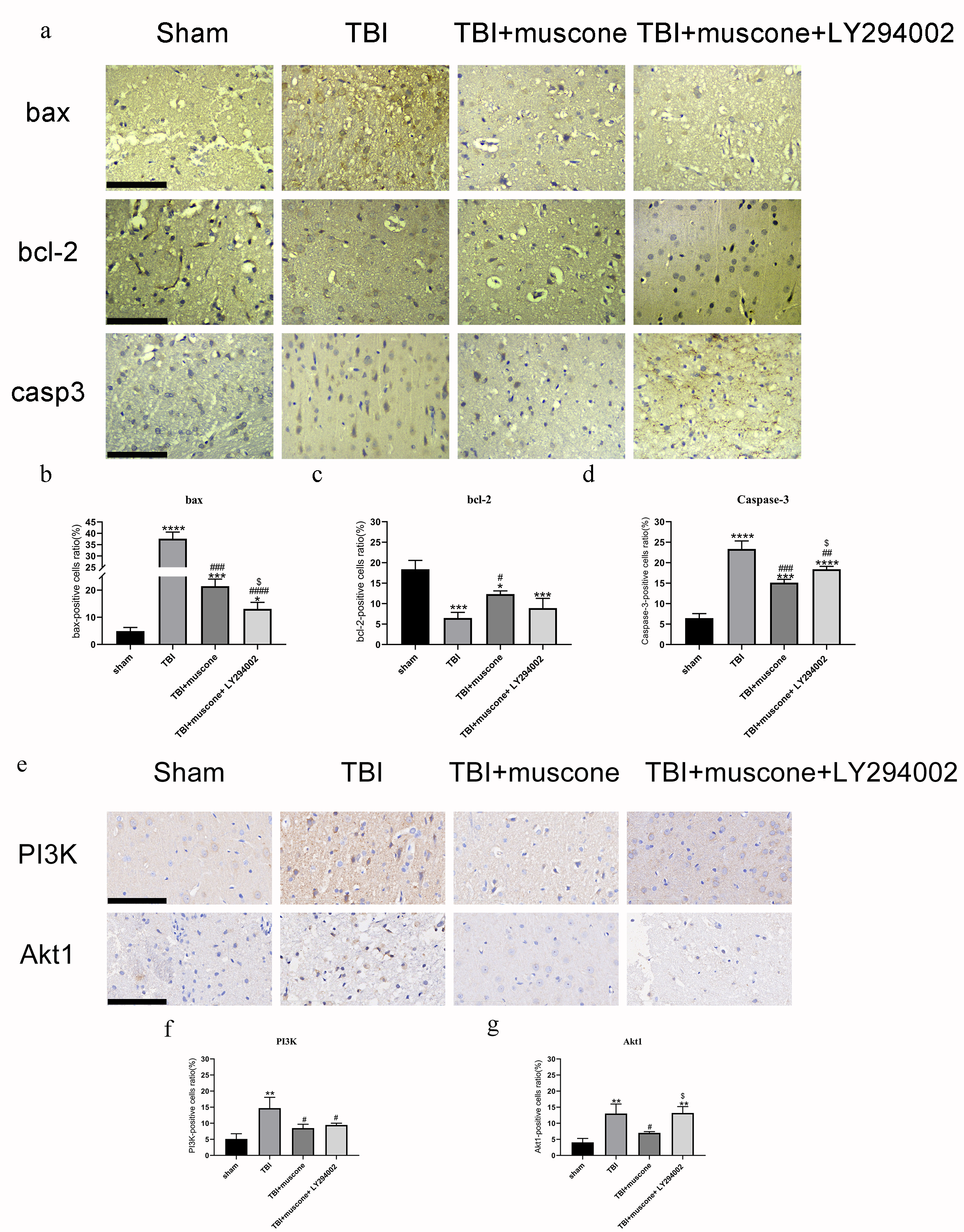

Supplement: Supplemental Information 3 — (A and E) Representative images of immunohistochemical staining for bax, BCL-2 , caspase-3,PI3K and Akt(scale bar = 100 um). (B–D, F and G) Quantification of immunohistochemistry results. N = 3,each group ∗P < 0.05, ∗∗P < 0.01, ∗∗∗P < 0.001, ∗∗∗∗P < 0.0001 vs sham group; #p < 0.05, ##p < 0.01, ###p < 0.001, ###p < 0.0001 vs TBI group; p < 0.05 vs TBI+muscone group. [file peerj-09-11696-s003.png]
