# Supplementary material for: Network pharmacology to investigate the pharmacological mechanisms of muscone in Xingnaojing injections for the treatment of severe traumatic brain injury
Source: PeerJ. 2021 Jul 20;9:e11696. doi: 10.7717/peerj.11696 (PMC8300495; doi:10.7717/peerj.11696)
Supplement: Supplemental Information 4 [file peerj-09-11696-s004.docx]

**Method of molecular docking**

**Molecular structure’s preparation**

We obtained and downed the SDF file of the molecule from PubChem database. Then, we used the Chem 3D software to optimize and save it(Xue-Dong, Gao & Zhang, 2017). Subsequently, the structure was imported into Schrodinger to establish the database. Finally, it was stored as the ligand after hydrogenating, structure optimizing, energy minimizing.

**Protein preparation**

The AKT1 of the protein crystal structure (PDB ID: 4GV1, Resolution: 1.49 Å) was downloaded from the protein database (https://www.rcsb.org/structure/4P1U). Then it was processed on the Maestro11.9 platform using Schrodinger’s The Protein Preparation Wizard including removing crystal water, adding missing hydrogen atoms, repairing missing peptides, minimizing the protein energy and optimizing the geometric structure(Rajeswari, Santhi & Bhuvaneswari, 2014; Fazi et al., 2015).

**Analysis of docking results**

We used the Glide module of Schrödinger Maestro to process and optimize the virtual screening(Software & Com). The action model of the positive control compound and Akt1 was analyzed to obtain the interaction with each residue, such as hydrogen bond interaction, π-π interaction, hydrophobic interaction, etc.Then rating the docking status of the compound to infer whether the small molecule has an active effect.

**Results of molecular docking**

Muscone has a high degree of matching with the active site of AKT1 protein and lower binding energy (-5.48 kcal/mol). The binding sites include PHE-438, PHE-442, MET-281, MET-227, VAL-164 amino acids Residues. The more negative the binding energy, the more plausible the interaction（when binding energy<-5 kcal/mol）. Due to high hydrophilicity, muscone can form strong hydrophobic interactions with the hydrophobic amino acids in the protein pocket, which has a greater effect on the stability of small molecules in the protein active pocket. Additionally, muscone can form weak hydrogen bonds with amino acid MET-281.These interaction forces can effectively improve the stability of the compound in the active pocket of the Akt1 protein.Therefore, the compound is a potentially active small molecule.


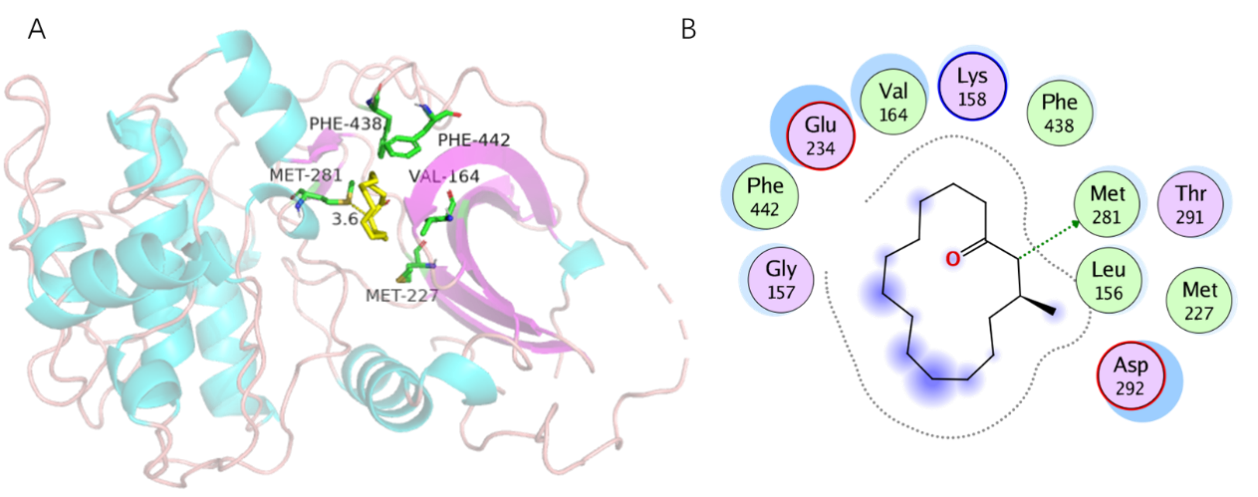


Figure S4 The binding mode of muscone with Akt1 target protein. (a)The binding mode of 3D structure.(b) The binding mode of 2D structure.

**Reference**

Fazi R, Tintori C, Brai A, Botta L, Selvaraj M, Garbelli A, Maga G, Botta M. 2015. Homology Model-Based Virtual Screening for the Identification of Human Helicase DDX3 Inhibitors. *Journal of Chemical Information and Modeling* 55:2443–2454. DOI: 10.1021/acs.jcim.5b00419.

Friesner RA, Banks JL, Murphy RB, Halgren TA, Klicic JJ, Mainz DT, Repasky MP, Knoll EH, Shelley M, Perry JK, Shaw DE, Francis P, Shenkin PS. 2004. Glide: A New Approach for Rapid, Accurate Docking and Scoring. 1. Method and Assessment of Docking Accuracy. *Journal of Medicinal Chemistry* 47:1739–1749. DOI: 10.1021/jm0306430.

Rajeswari M, Santhi N, Bhuvaneswari V. 2014. Pharmacophore and Virtual Screening of JAK3 inhibitors. *Bioinformation* 10:157–163. DOI: 10.6026/97320630010157.

Software S, Com S. Schrödinger Molecular Modeling Software - Northeastern ITS.

Xue-Dong WU, Gao SZ, Zhang F. 2017. The Application of Chemdraw Software in Chemistry of Dyestuffs Teaching. *Heilongjiang Textile*:4–6.
